# Mechanochemical Depolymerization of PET: Kinetic Studies on Alkaline Hydrolysis of Commercial Feedstocks

**DOI:** 10.1002/cssc.202502416

**Published:** 2026-02-12

**Authors:** Kinga Gołąbek, Lauren R. Mellinger, Shanell T. Bush, Erin V. Phillips, Georgios A. Marinis, Van Son Nguyen, Jouke van Westrenen, Carsten Sievers

**Affiliations:** ^1^ School of Chemical & Biomolecular Engineering Georgia Institute of Technology Atlanta Georgia USA

**Keywords:** ball milling, crystallinity, feedstock morphology, polymers, reactive interfaces

## Abstract

The mechanochemical depolymerization of commercial PET feedstocks is successfully demonstrated for a variety of samples representing consumer products without the need for specific sample pretreatment. Complete depolymerization is achieved within 20 min by ball milling it with NaOH under ambient conditions. Samples with a higher initial content of amorphous domains depolymerize more rapidly, as collision energy is more effectively utilized for creating reactive interfaces between NaOH and PET. While thickness has a minor effect compared to crystallinity, thicker samples experience lower reaction rates because their accessible surface area is limited. For low‐packing density samples, a reduced rate of depolymerization could be expected due to restricted ball motion, but this effect is overcompensated by the ease at which these samples form interfaces. The success of mechanochemical alkali‐depolymerization of PET in a ball mill presents an opportunity for industrial implementation, offering a sustainable approach to polymer upcycling due to its mild reaction conditions and minimal solvent requirements.

## Graphical abstract

Complete depolymerization of commercial PET feedstocks in the presence of NaOH is achieved within 20 min under ambient conditions, without any specific sample pretreatment. Depolymerization is accelerated in samples with higher amorphous content due to more efficient interface formation. Although thickness plays a minor role, reduced surface area in thicker samples leads to slower reaction rates.

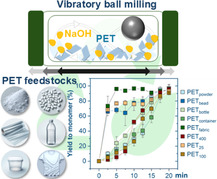

## 1 Introduction

Poly(ethylene terephthalate) (PET) is one of the most commonly used commercial plastics, with more than 82 million metric tons produced globally for use in packaging, clothing, and other industries [1]. The properties that make PET especially useful include its light weight, high durability and toughness, and resistance to carbon dioxide permeation, specifically for packaging applications [2]. However, PET is one of the largest sources of plastic waste because of its high volume of production worldwide. In 2023, the PET recycling rate in the US was only 33%, despite the increased production of PET for commercial use [3]. Additionally, PET poses a significant environmental concern since it takes around 300 years to fully decompose [4]. Consequently, it is important to explore environmentally friendly recycling methods to reduce PET pollution.

The most common recycling methods used for commercial PET feedstocks are mechanical and chemical recycling, though they both have their own unique challenges [5, 6]. Large‐scale mechanical recycling requires separation of any contaminants through various techniques such as washing, mechanical agitation, or thermal treatment [7, 8]. A study concluded that both the number of contaminants and the type of contaminants in the PET feed stream played a critical role in the yield to monomer, highlighting one of the major flaws with mechanical recycling [9]. Another significant shortcoming of mechanical recycling is a reduction in the quality and increased brittleness of the PET monomers produced due to repeated crushing and cutting of PET [8, 10]. Additionally, detailed regulations set by the FDA for the mechanical recycling of food and drug packaging hinder the applicability of this method due to contamination concerns [11]. Chemical recycling is attractive because of its potential use for closed‐loop recycling and its high product purity. In particular, hydrolysis of PET into ethylene glycol (EG) and Terephthalic acid (TPA) can be conducted in acidic, basic, or neutral reaction conditions from 200°C to 250°C [12]. However, hydrolysis under highly acidic or basic conditions can be corrosive to process equipment and is consequently an economically unfavorable process [12, 13]. In addition, recovering high‐purity TPA from the hydrolysis reaction requires advanced energy‐intensive separation techniques. Glycolysis is an alternate chemical depolymerization method, which can achieve a 70% yield to monomer after an hour of reaction time [10] but requires a variety of solvents, including zinc acetate, sodium carbonate, sodium bicarbonate, sodium sulfate, and potassium sulfate [10]. Finally, alcoholysis of PET using multi‐functional catalysts like polyoxometalates can completely degrade PET in five minutes, and successful catalysts have been reused more than 30 times. However, similarly to glycolysis, catalyzed alcoholysis requires both harsh operating conditions and extensive solvent usage, reducing the greenness of the process [14]. The limitations of existing mechanical and chemical recycling techniques for recycling commercial PET feedstocks highlight the potential for an improved process requiring minimal pretreatment, moderate reaction conditions, and short reaction times, with mechanochemistry as a promising alternative.

Mechanochemistry refers to chemical reactions driven by mechanical energy, such as the impact of grinding balls in a milling process. This approach has gained attention due to its minimal solvent usage, reduction of the overall energy consumption, and increase in the sustainability of recycling [15]. Successful mechanochemical depolymerization of PET pellets to disodium terephthalate (Na_2_TPA) and ethylene glycol (EG) has been observed by milling in the shaker mill at 30 Hz with a stochiometric ratio of sodium hydroxide crystals at ambient temperatures for 20 min [16, 17]. This depolymerization process is characterized by a phase transition from a fine powder to a wax at around 40% conversion, which causes a significant increase in the yield to monomer. Additionally, the reaction kinetics can be accurately explained by a modified shrinking core model, wherein the interfacial contact introduced by the collisions is the limiting factor in the kinetics instead of the reactant diffusion. In particular, the milling intensity was used as an energetic descriptor and was found to be linearly proportional to the reaction rate. This kinetic model was successfully validated by plotting the kinetic energy of the ball and collision frequency as a function of the reaction rate, where both parameters were proportional to the milling intensity [16]. These kinetics contrast with PET synthesis via step polymerization, which is characterized by rapid consumption of monomers before production of polymers [18]. Recent studies highlight the broader relevance of mechanochemical plastic recycling, with several studies demonstrating efficient solvent‐free depolymerization of diverse condensation polymers under mild conditions and without catalysts, highlighting solid‐state ester cleavage across polymer classes [19, 20]. Ball milling has emerged as a robust platform for chemical recycling, capable of overcoming solubility constraints, reducing energy requirements, and achieving high monomer selectivity under conditions that are inaccessible to traditional thermochemical approaches [21].

Along with successful mechanochemical depolymerization of PET at ambient operating conditions on a lab scale, a promising technoeconomic analysis was conducted for the process [22, 23]. Process simulation predicted 97% monomer recovery with 99%+ purity, along with “zero‐liquid discharge” due to the complete recovery of ethylene glycol and recycling of process water [22]. However, this process has not been extrapolated to commercial forms of PET, which are not pure and may require initial treatment before recycling. Moreover, the physical properties of specific forms of PET could have a significant effect on the kinetics of the mechanochemical depolymerization reaction.

In this study, the mechanochemical depolymerization of different commercial and post‐consumer PET feedstocks is studied. For each of the feedstocks, the kinetic behavior is characterized along with the impact of feedstock properties during depolymerization, such as crystallinity, shape, and external surface area. This process shows great potential for effective industrial recycling applications because of its ambient milling temperatures and pressures, short reaction time, and minimal solvent use.

## 2 Results

Since it has been established that PET depolymerization with NaOH is limited by the formation of reactive interfaces between the reactants [16], kinetic expressions for different feedstocks were expected to strongly depend on their structure and/or morphology. To study the feedstock specific kinetics of mechanochemical alkali‐depolymerization of commercial PET samples, six different PET feedstocks were used: powder (PET_powder_), beads (PET_beads_), films (PET_400_, PET_100_ and PET_25_) of varying thickness (denoted per thousandth of an inch in subscripts), a bottle (PET_bottle_), a food container (PET_container_), and fabric (PET_fabric_). The PET powder, beads, and films had well‐defined physical properties, while the bottle, food container, and fabric were all post‐consumer plastic products without detailed physical specifications. To analyze the depolymerization and kinetic behavior of these feedstocks, two distinct groupings were made based on dimensions and shape. The first was the particle‐like samples, which included the powder and beads. The PET powder was additionally pre‐milled prior to depolymerization to reduce its crystalline content. This pre‐treated sample is hereafter referred to as PET_pre‐milled_. The second grouping was the flake‐type samples, consisting of all three PET films, bottle, food container, and fabric. The second group of samples was all cut into small flakes of roughly the same size before addition to the ball mill and were thus considered to be similar sample types.

### 2.1 Textural Analysis of Commercial Feedstocks

**FIGURE 1:**
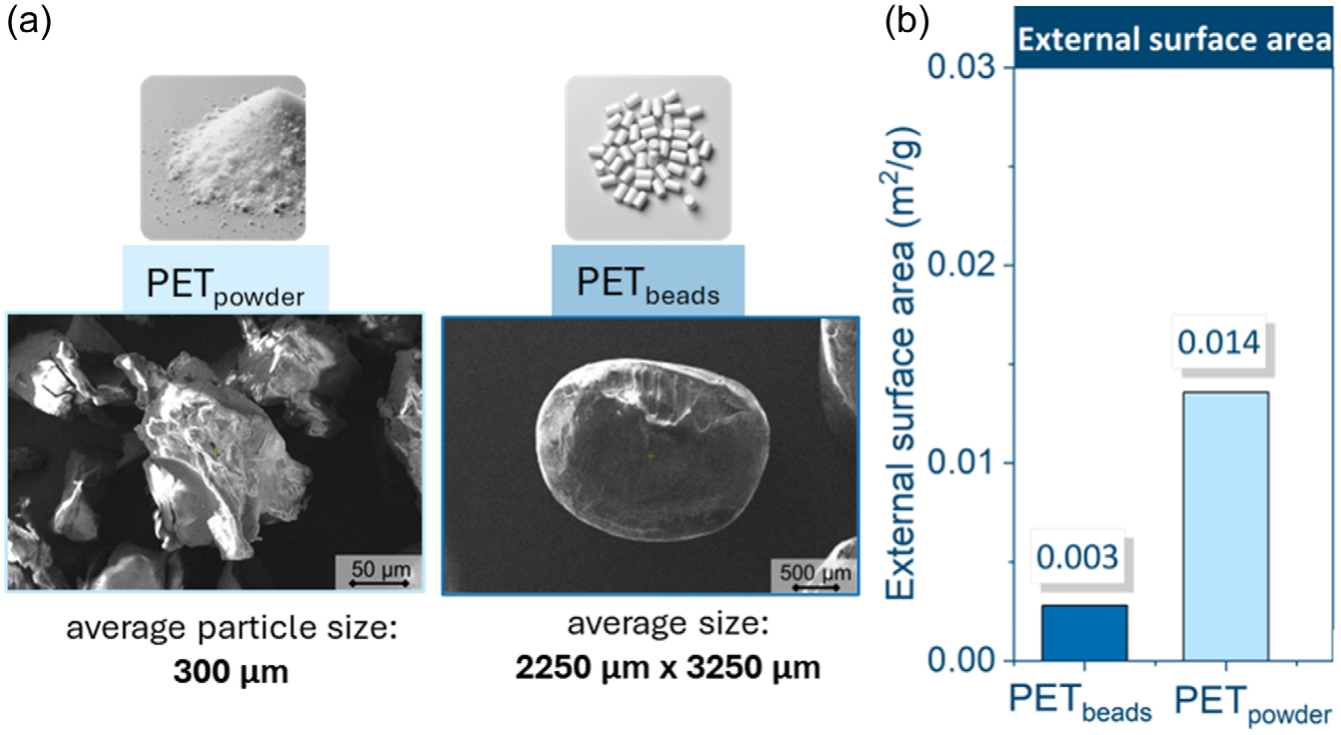
SEM images of powder and beads, with average particle size values (a) and external surface area calculated according to the equations presented in the experimental section (b).

Scanning electron microscopy (SEM) images of PET_powder_ particles exhibited an irregular shape with rough edges and an average diameter of 300 μm (Figure 1a). In contrast, PET_beads_ were cylindrical with smooth edges, measuring approximately 2250 by 3250 μm, nearly 100 times larger than the powder particles. Additionally, PET_powder_ exhibited a noticeably higher density (1.472 g/cm^3^) compared to PET_beads_ (1.395 g/cm^3^), indicating a more compact microstructure. Consistent with these parameters, PET powder had an external surface area nearly five times greater than that of PET_beads_ (Figure 1b). Given the shape and dimensions of these samples, the calculated vessel occupancy volumes were 2.5 cm^3^ for PET_powder_ and 2.0 cm^3^ for PET_beads_, as indicated in Table SI1 of the Supplementary Information.

Based on these values, the movement of the samples within the milling vessel can be considered free‐flowing. The primary takeaway from this size comparison is that PET beads are significantly larger than PET powder, suggesting that any variations in depolymerization behavior may be attributed to this size disparity.

**FIGURE 2:**
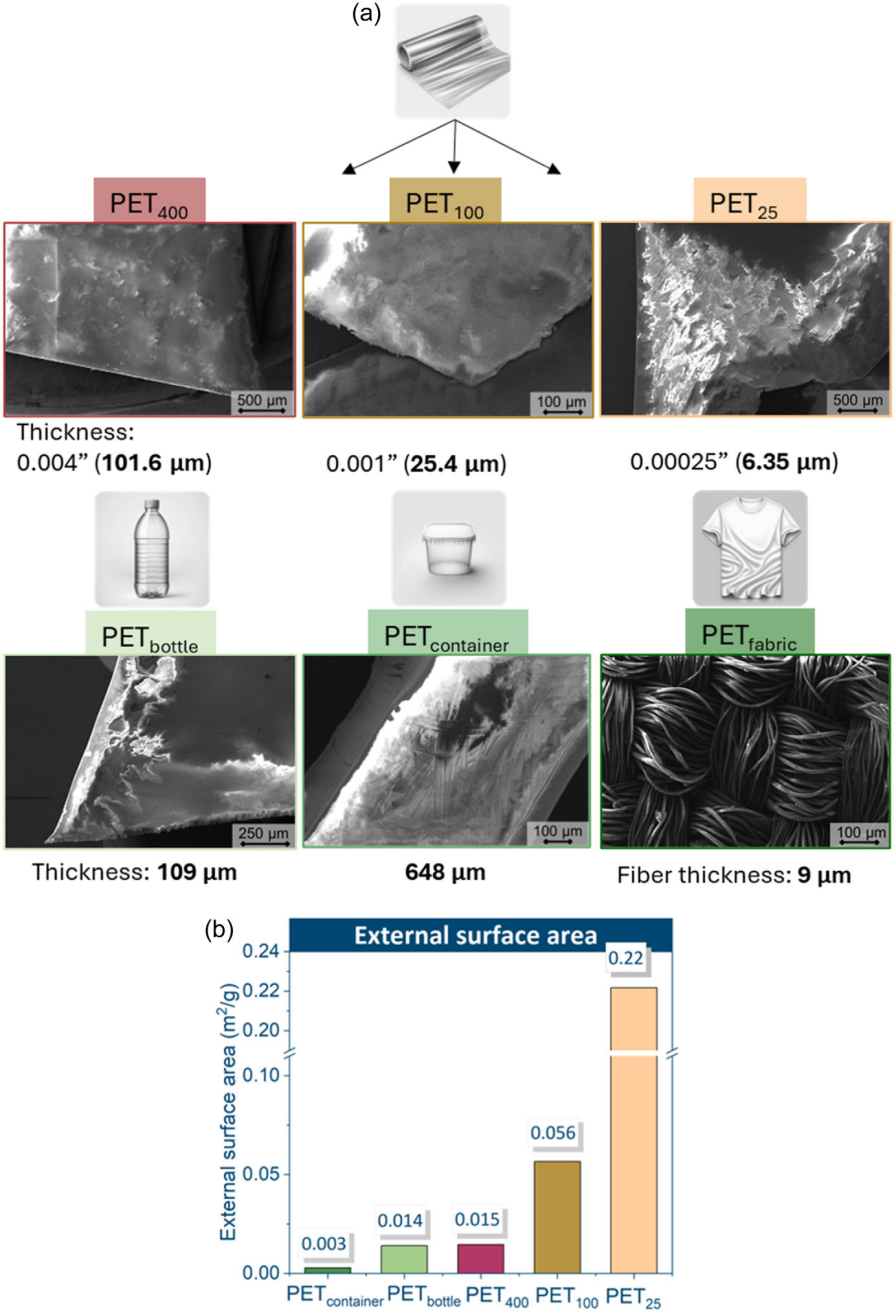
SEM images of flake‐type PET samples, with thickness values (a) and external surface area (b).

The top‐view morphology of the flake‐type samples was mostly featureless (Figure 2a). The studied samples possessed a compact structure, and no visible particle‐like contaminants were observed. The distinct difference between the flakes was their thickness. The texture of PET_container_ exhibited a lamellar structure, as well as some apparent texture of striations that were reminiscent of stress due to mechanical forces [3]. The reduced quality of the SEM images captured for flake‐type samples is attributed to the high surface resistance [24] of PET, which favors the buildup of static charge during the capture of the micrographs. The accumulated charge can saturate the detector, leading to the appearance of bright spots in the SEM images [25, 26]. The SEM analysis was particularly useful for characterizing the structure of the fabric. The image showed a tightly woven fabric structure. The individual fibers were clearly visible and appeared to be relatively smooth, densely packed together, and cylindrical in shape, measuring approximately 9 μm, which is characteristic of synthetic fibers like polyester [27, 28].

Among all flake‐type samples, the densities fell within a relatively tight range (1.337–1.509 g/cm^3^; Table SI5), with PET_fabric_ showing the highest value (1.509 g/cm^3^) likely due to its oriented fiber structure (as supported by the X‐ray diffraction (XRD) analysis presented in the next section), while PET_container_ showed the lowest density (1.337 g/cm^3^), reflecting typical variations in commercially processed PET.

The thickness of each of the PET films and post‐consumer flakes was measured using an electronic caliper. These values are shown in Figure 2a, along with the external surface area of the different feedstocks ranked from thickest to thinnest in Figure 2b. An important finding was that PET_400_ and PET_bottle_ had roughly the same thickness, meaning that the depolymerization behavior of these samples can be easily compared without the influence of size on potential yield variances to monomer. The food container was six times thicker than the bottle and more than 100 times thicker than the thinnest film (PET_25_). The PET_container_ had the same surface area as the PET_beads_. The average external surface area of the films inversely correlated with the average film thickness, with PET_25_ having the greatest surface area and PET_container_ the lowest.

Lastly, the sample occupancy vessel volumes were computed for all flake‐type samples and decreased in the following order: PET_25_ > PET_fabric_ > PET_100_ = PET_bottle_ > PET_400_ > PET_container_. Images depicting vessel occupancy, along with the methodology and numeric occupancy values, can be found in the Supplementary Information in Table SI1 and Figure SI1a,1b. Considering that the PET_bottle_ and PET_400_ have the same thickness but different vessel occupancies, the PET_bottle_ has a lower packing density than PET_400_ and fills a greater volume of the vessel. Additionally, the sample volume was greater for PET_100_ and PET_25_ compared to PET_powder_, indicating that the free‐flow movement of these flakes may be limited during the milling process due to high vessel occupancy.

### 2.2 Microstructural Analysis of Commercial Feedstocks

**FIGURE 3:**
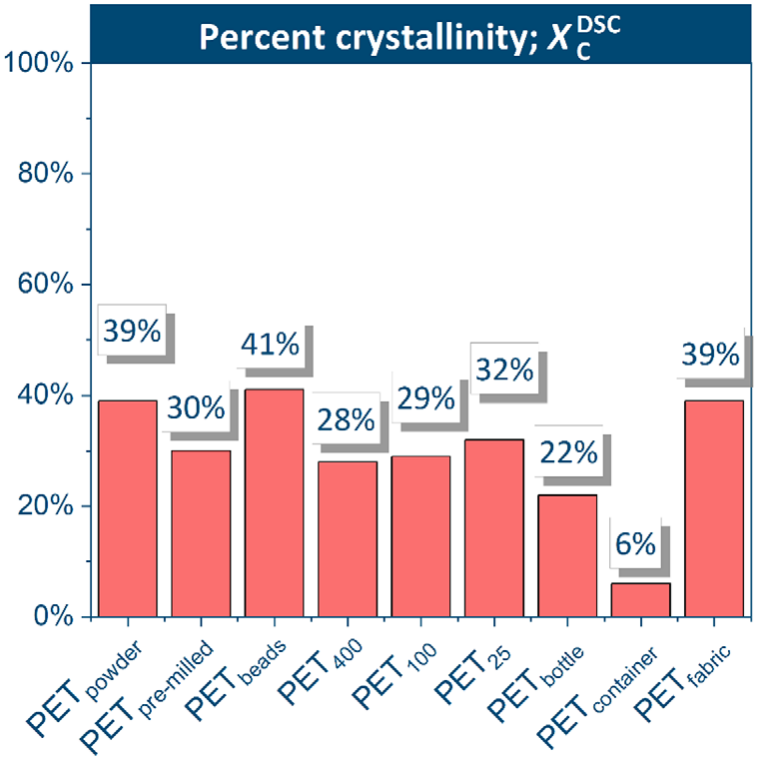
Bulk percent crystallinity (*X* *_C_* ^DSC^) calculated for all PET samples.

To quantify the structural differences among the various commercial PET feedstocks, the bulk percent crystallinity (*X* *_C_* ^DSC^; Figure 3) obtained from differential scanning calorimetry (DSC) measurements (Figure SI4–SI11) was first examined. Bulk crystallinity refers to the overall crystalline fraction within the material, averaged over the entire sample rather than localized surface or domain‐level features. The bulk crystallinity followed the order: PET_beads_ (41%) > PET_powder_ ≈ PET_fabric_ (each 39%) > PET_25_ (32%) > PET_pre‐milled_ (30%) > PET_100_ (29%) > PET_400_ (28%) > PET_bottle_ (22%) > PET_container_ (6%). On the other hand, surface crystallinity describes the ordering confined to the near‐surface region, which can differ markedly due to processing history, thermal gradients, or mechanical deformation [29].

In PET, particularly films and fibers, surface crystallinity has been reported to deviate from bulk values, and in some cases to exceed them, reflecting enhanced chain orientation or localized recrystallization during manufacturing [29, 30]. Considering that surface ordering may not necessarily track with bulk crystallinity, the surface crystallinity was also evaluated.

**FIGURE 4:**
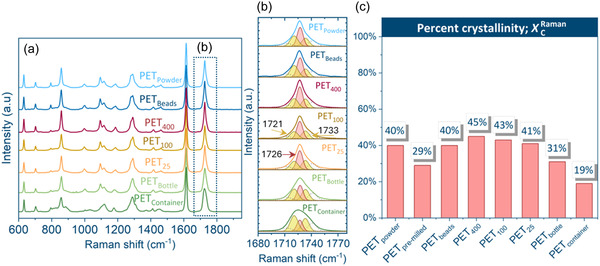
Raman spectra of PET samples (a); The band corresponding to the crystalline (red) and amorphous (yellow) conformers, as revealed by peak fitting analysis (b); The surface percent crystallinity (*X_C_* ^Raman^) from the Raman analysis of the C = O stretching band using the peak fitting procedure (c).

The Raman spectra were collected for each of the samples, and surface percent crystallinity (*X* *_C_* ^Raman^) was calculated using the method proposed by Bouita et al [31]. The peak at 1727 cm^−1^ is assigned to stretching modes of the C = O groups of terephthalate units that are coplanar with the benzene ring, and glycol units are in *trans* conformation, which is the dominant configuration in crystalline domains. In contrast, the peaks at 1721 and 1733 cm^−1^ are attributed to C = O groups in gauche formation, which is representative of amorphous phases [32, 33]. The area under these characteristic bands was used to calculate surface percent crystallinity (*X* *_C_* ^Raman^) in the starting feedstock material, which is shown in Figure 4c below.

The multiplicity of bands with overlapping positions was noted in the acquired Raman spectra of all studied samples (Figure 4a). All the characteristic vibrations corresponding to PET terephthalate as well as ethylene glycol units were identified; all band positions matched those reported in the literature [34]. Raman spectra of all post‐consumer samples are nearly identical to the PET_powder_, implying that if there are any additives in the post‐consumer samples, they are present in such small quantities that they are below the detection limit. The particle‐like samples, PET_powder_ and PET_beads_, had identical surface percent crystallinity (40%). Since there was no difference in crystallinity, the particle size difference between these samples was very large. It is concluded that the governing factor to explain any difference in reaction behavior may be the particle shape and size.

For the flake samples, there were some notable differences in surface percent crystallinity, which can be ranked in the following order: PET_400_ (45%) > PET_100_ (43%) > PET_25_ (41%) >> PET_bottle_ (31%) > PET_pre‐milled_ (29%) > PET_container_ (19%). The three PET films had a similar abundance of amorphous conformers despite their differences in thickness, which is likely attributed to the similar manufacturing process of these films. These samples had a slightly higher fraction of crystalline conformers than the particle‐like samples. It is interesting to note that the samples with the highest population of amorphous regions were the food container and bottle, which are both post‐consumer plastics.

In the case of the post‐consumer flake‐type samples, the maximized number of amorphous domains in the PET structure made the samples highly transparent, which is a favorable quality for PET consumer goods. To manufacture transparent bottles and containers, PET is cooled rapidly during the manufacturing processes, which prevents crystalline structure formation and results in predominantly amorphous material [35, 36]. This phenomenon accounts for the greater amorphous character observed in post‐consumer feedstocks, both at the surface and in the bulk, compared with the highly ordered lab‐grade PET films. Although the overall trends in bulk and surface crystallinity were broadly comparable, all flake‐type samples showed meaningful differences between *X* *_C_* ^DSC^ and *X* *_C_* ^Raman^, consistent with prior studies that PET can exhibit surface‐specific ordering distinct from its bulk structure [29, 30]. These differences highlight that bulk crystallinity alone is insufficient to fully describe the structural properties relevant to mechanochemical depolymerization, thereby motivating the parallel evaluation of surface crystallinity.

**FIGURE 5:**
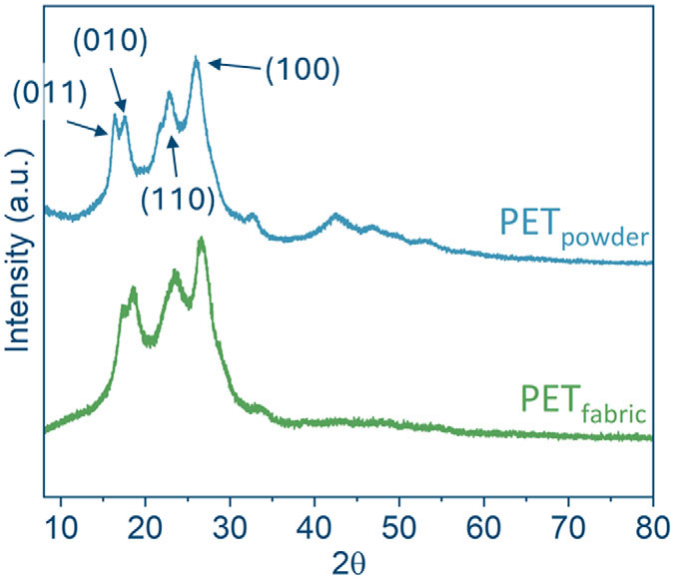
XRD patterns collected for PET_powder_ and PET_fabric_.

Given the challenges of Raman analysis of the dyed fabric sample, its surface crystallinity was assessed at the domain level using XRD (Figure 4) [37, 38]. PET_powder_ served as the reference sample. A broad amorphous halo centered at approximately 2*θ* ≈ 23° on the *x*‐axis of Figure 5 was visible in both samples. Additionally, characteristic diffraction peaks of crystalline PET were distinguished at 2*θ* = 16.4°, 17.6°, 22.5°, and 26.2°, corresponding to the (011), (010), (110), and (100) lattice planes[39]. The (100) diffraction peak is often enhanced in oriented samples, such as uniaxially drawn PET fibers and films. However, due to the prominent amorphous halo in both samples, direct peak height comparison was challenging. Because of these limitations, surface crystallinity could not be reliably determined for the fabric sample. For consistency across all feedstocks, the bulk crystallinity of PET_fabric_ obtained from DSC was therefore used for further analysis.

Additionally, the ratio of the (100) peak height to the sum of all distinct diffraction peak heights was calculated. This ratio, denoted here as *f*_XRD_, is commonly interpreted as an orientation factor [40] rather than a quantitative measure of absolute crystallinity. The calculated values of *f*_XRD_ were 1.5 for PET fabric and 0.7 for PET powder, confirming a higher degree of uniaxial orientation in the commercial PET fabric.

### 2.3 Mechanochemical Alkali‐Depolymerization: Progression of the Reaction

During the milling process, PET reacts with two equivalents of sodium hydroxide to depolymerize and form two monomers in an equimolar ratio, ethylene glycol (EG) and disodium terephthalate (Na_2_TPA). Specifically, the yield was determined by high‐performance liquid chromatography (HPLC) following the method of Tricker et al. [16]. The reaction mixture was dissolved in water, serially diluted, and filtered prior to analysis. A full description of the sample preparation protocol and the calculation of monomer yield (Equation (9)) is provided in the Experimental Section. For simplicity, the extent of reaction is reported in terms of the yield of Na_2_TPA.

**FIGURE 6:**
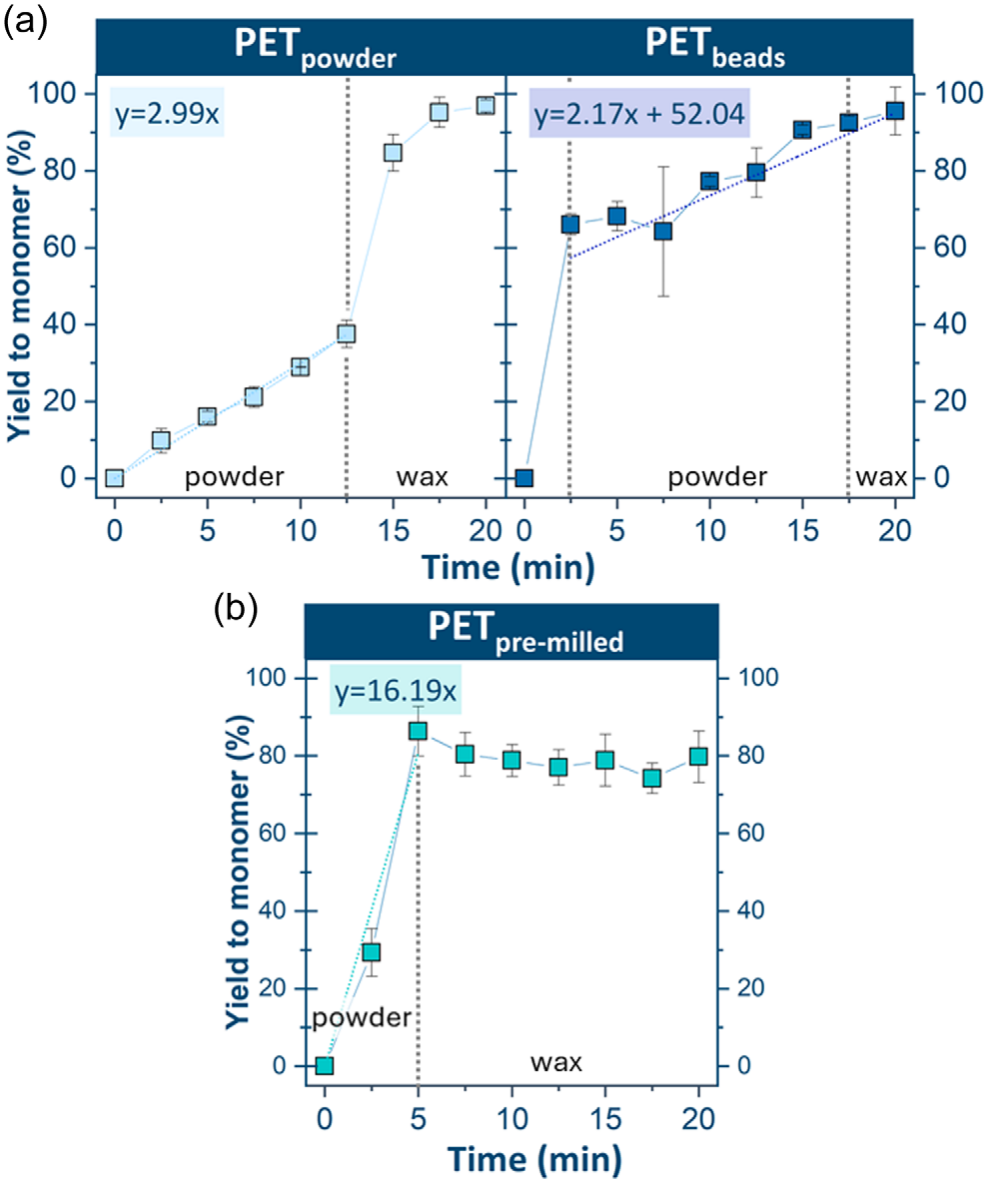
Na_2_TPA monomer yield over time for PET_powder_ and PET_beads_ (a) and pre‐milled powder (PET_pre‐milled_) (b).

The behavior of the PET_powder_ was measured and used as the baseline for analysis of both the particle‐like and flake‐type samples [16]. For PET_powder_, the yield to monomer increased linearly until 12.5 min, when a phase transformation from a powder into homogeneous wax occurred, and the rate of monomer formation accelerated (Figure 6a) as described in earlier work [16]. However, this phenomenon was not observed in the case of the beads. A high monomer yield was already observed after 2.5 min (Y_Na2TPA_ = 66%), but the feedstock mixture did not become completely waxy (Figure SI2) until around 17.5 min when monomer yield was 95%. Thus, the feedstock phase transformation was not correlated to the spike in the monomer yield as for powders [16]. Instead, there was a linear increase in monomer yield from PET_beads_, when the beads were in a powder state after 2.5 min. In contrast, the pre‐milled PET powder exhibited markedly different behavior compared to the parent PET powder (Figure 6b). A rapid increase in Na_2_TPA yield was observed within the first 5 min, reaching approximately 85%, during which the milling feedstock transformed from a powder into a homogeneous wax. The monomer yield subsequently remained relatively constant despite continued milling, without reaching complete depolymerization.

### 2.4 Flake‐Type Samples

**FIGURE 7:**
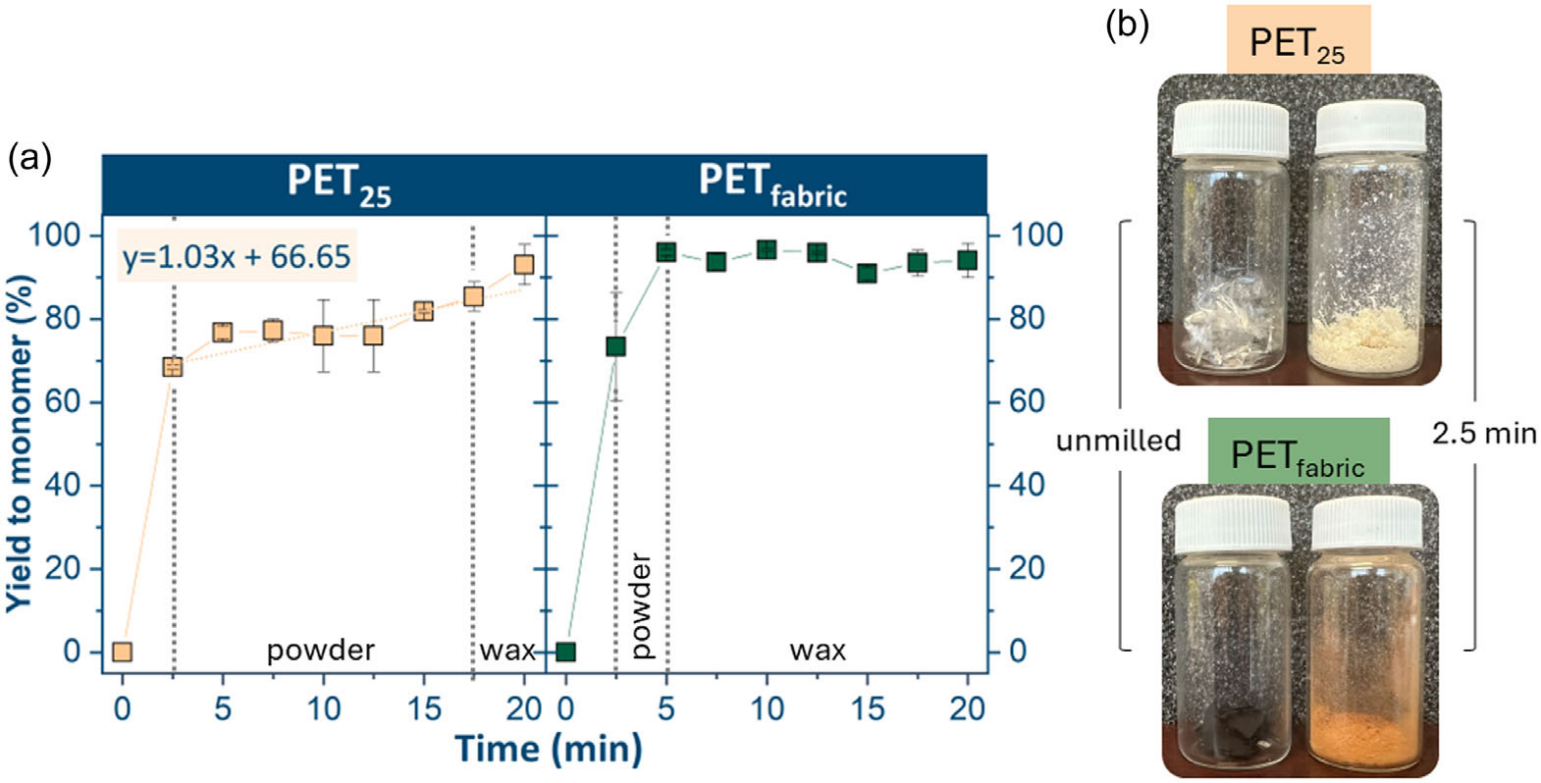
Na_2_TPA monomer yield over time for PET_powder_ and PET_beads_ (a) and images of milling feedstocks before and after milling for 2.5 min (b).

**FIGURE 8:**
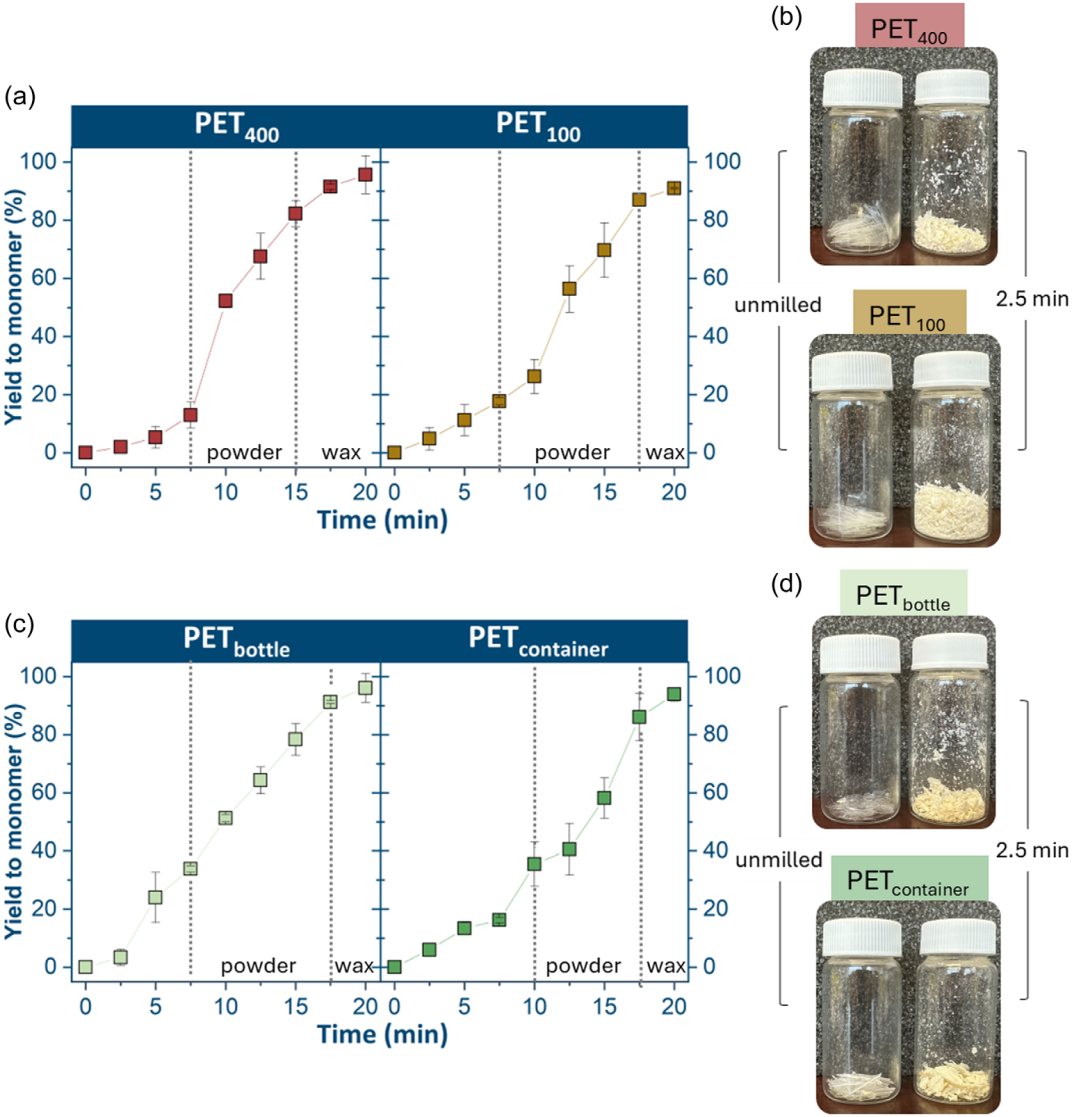
Na_2_TPA monomer yield over time for PET_400_ and PET_100_ (a); images of PET_400_ and PET_100_ before and after milling for 2.5 min (b); Na_2_TPA Monomer yield over time for PET_bottle_ and PET_container_ (c); images of PET_bottle_ and PET_container_ before and after milling for 2.5 min (d).

The depolymerization behavior of the flake‐type samples is illustrated in Figures 7 and 8. The PET_25_ and PET_fabric_ behaved noticeably differently compared to the other four feedstocks. Despite variations in shape and texture, the depolymerization progression curves for both PET_25_ and PET_fabric_ were strikingly similar (Figure 7). A high initial rate of reaction was observed for both samples, with values reaching 68% (PET_25_) and 73% (PET_fabric_) at 2.5 min for both samples, which appeared as powder. The observed color change in the fabric after 2.5 min was likely due to the chemical transformations of the pigment upon collision; however, a detailed analysis of this transformation falls outside the scope of this study.

In the subsequent 2.5 min, PET_fabric_ approached nearly 100% monomer yield, with the milling feedstock transitioning from powder to a waxy state, as the depolymerization was completed. This unusually early phase transition is consistent with the rapid formation of EG (liquid) and Na_2_‐TPA (fine crystals), which together generate a compacted, waxy mixture under milling. Since PET_fabric_ depolymerizes significantly faster than the other samples, the critical monomer concentration required to induce wax formation is reached much earlier. In contrast, the increase in monomer yield for PET_25_ was more gradual. Over the 20 min of the reaction, the monomer yield for PET_25_ increased at a constant rate and ultimately did not achieve 100% yield to monomer. Notably, the homogenous waxy phase for PET_25_ emerged only at 17.5 min into the reaction.

The remaining four flake‐type samples (PET_bottle_, PET_400_, PET_100_, and PET_container_) showed broadly similar monomer yield profiles, though with notable differences in the presence and sharpness of inflection points (Figure 8). PET_400_ and PET_100_ displayed a clear inflection point, after which the monomer formation rate increased substantially. In contrast, PET_bottle_ exhibited an almost linear increase in monomer yield throughout the milling period, without a pronounced inflection. PET_container_ showed a distinct inflection point at 10 min, coinciding with its full pulverization, followed by a more moderate increase in monomer yield. The transition from powder to wax phase occurred at 15 min for PET_400_ and at 17.5 min for PET_bottle_, PET_100_, and PET_container_. Although PET_400_ and PET_bottle_ ultimately achieved 100% yield within experimental error, PET_100_ and PET_container_ reached slightly lower maximum yields of ∼92% and ∼95%, respectively. These slightly lower final yields likely arise from differences in feedstock composition, rather than from differences in thickness alone.

One initially noticeable feature was that none of these samples were pulverized in the first 2.5 min (Figure 8b,d). In the initial stage, they appeared as a heterogeneous mixture of powder and flakes, as shown in the images. Additionally, PET_400_ and PET_100_ had a clear inflection point correlating closely with the wax phase transition, implying that there is a notable distinction in the PET_400_ and PET_100_ samples compared to the post‐consumer samples, leading to the slow initial rise in yield to monomer.

It is noticeable that the wax phase appeared at different time points for the various feedstocks. It seems that wax forms once sufficient ethylene glycol and crystalline Na_2_‐TPA are generated at high monomer yields, promoting particle adhesion. These conditions were reached at different times for different feedstocks; their transition into the wax phase also occurred at different stages. The uniform powder developed wax early, whereas more heterogeneous samples, such as PET_25_ and PET_100_, formed wax later despite achieving similar monomer yields.

Given that even a single collision results in the formation of additional amorphous PET conformers [41], the surface percent crystallinity was quantified (Table SI5) for all milled samples at various time intervals, and its influence is discussed in detail in the Discussion section.

### 2.5 Kinetic Studies of Alkali‐Depolymerization

Initial rates of monomer formation were determined from plots of concentration against the time. The graph (Figure SI3) can be found in the Supplementary Information.

The linear correlation between monomer concentration and time during the first 12.5 min of the PET_powder_ depolymerization (Figure 6) pointed out that the reaction was zero‐order, and the rate constant depended strictly on the amount of energy supplied and structural characteristics of the feedstock instead of changes in bulk concentration.

The PET_powder_ was taken as a reference for the kinetic study with a rate constant of 1.25  ×  10^−4^ mol/dm^3^•s, while the pre‐milled PET powder showed higher rate constant of 6.71 × 10^−4^ mol/dm^3^•s The PET_400_ and PET_100_ both fit the kinetic model described in the Kinetic Model Calculations section in the Supporting Information accurately, with reaction rates of 6.3 × 10^−5^ mol/dm^3^•s and 1.05 × 10^−4^ mol/dm^3^•s, respectively (Figure SI3). The post‐consumer feedstocks also followed the linear kinetic model, with faster conversion to monomer rates than PET_400_ and PET_100_ of 2.07 × 10^−4^ mol/dm^3^•s (PET_bottle_) and 1.38 × 10^−4^ mol/dm^3^•s (PET_container_). The rates of PET_25_, PET_beads_, and PET_fabric_ were also computed using the linear fitting (1.14 × 10^−3^ mol/dm^3^•s, 1.1 × 10^−3^ mol/dm^3^•s, and 1.12 × 10^−3^ mol/dm^3^•s, respectively), despite potential overestimation from the calculation method (see Supporting Information). The ranking of the samples from slowest to fastest kinetic behavior is as follows, with *R* ^2^ values for the initial rates reported in the Supporting Information (Table SI3): PET_400_ < PET_100_ < PET_powder_ < PET_container_ < PET_bottle_ < PET_pre‐milled_ < PET_beads_ = PET_25_ = PET_fabric_.

## 3 Discussion

### 3.1 Influence of Feedstock Properties on Depolymerization Efficiency

While all PET samples were depolymerized with NaOH, the kinetics of PET depolymerization strongly depended on the type of PET feedstock. In conventional thermochemical liquid‐phase systems [42], hydrolysis mostly faces diffusional limitations, particularly in crystalline domains. Below the melting point of PET (245°C–265°C), the reaction relies on polymer chain mobility and permeability to proceed. Similarly, glycolysis is constrained by the initial raw material size and the presence of impurities [43], both of which hinder PET accessibility throughout the reaction.

**FIGURE 9:**
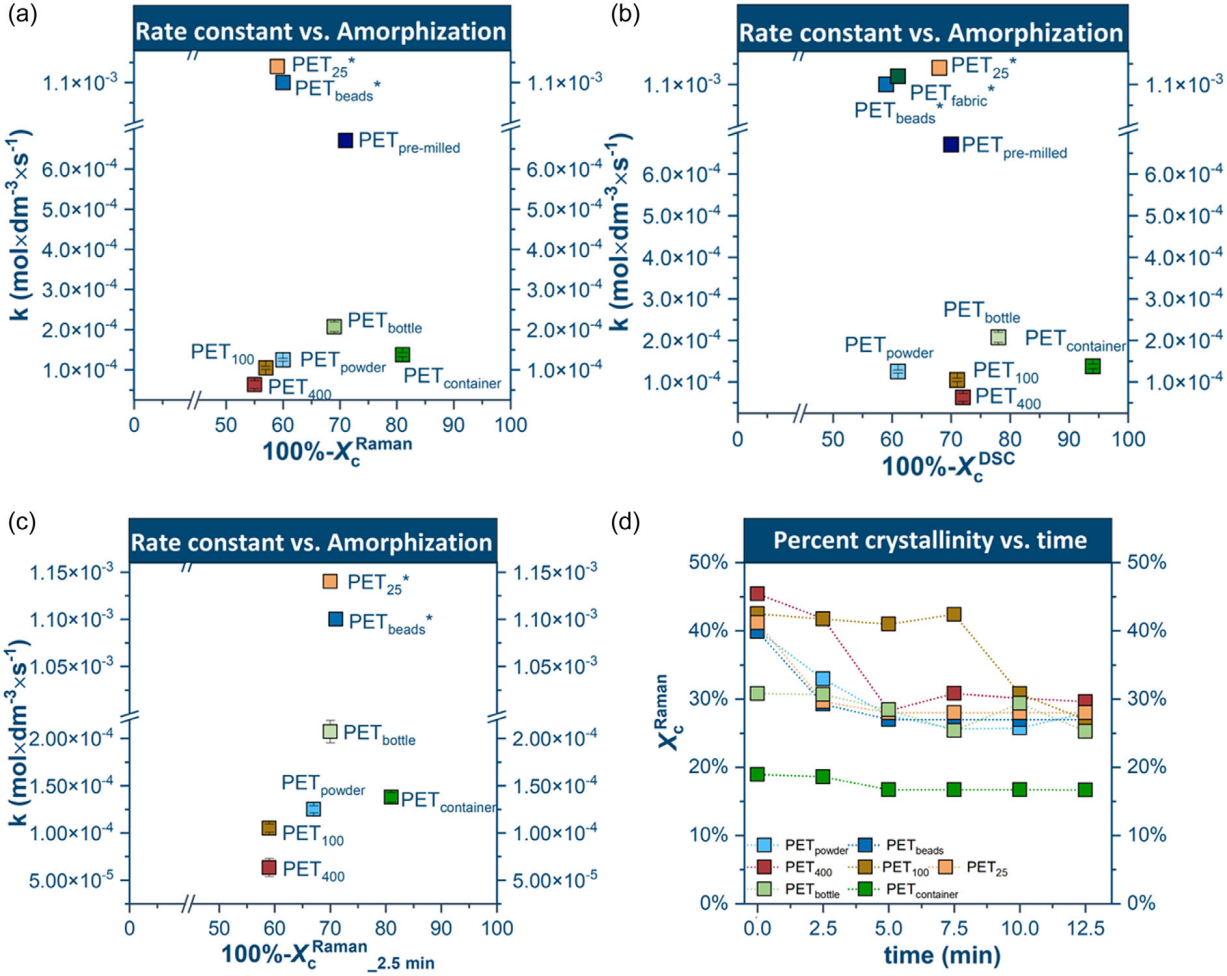
Correlation between reaction rate constant (*k*) and initial content of bulk amorphous phase expressed by 100%‐ *X* *_C_* ^DSC^(a) and surface amorphous phase expressed by 100%‐ *X_C_* ^Raman^ (b); Correlation between reaction rate constant and content of surface amorphous phase after milling for 2.5 min expressed by 100%‐ *X* *_C_* ^Raman^__2.5 min_ (c); Correlation between surface percent crystallinity (*X* *_C_* ^Raman^) over milling time. (d).

Crystalline domains need to be amorphized in the process of interface formation [41]. Amorphous domains, characterized by their loosely packed structure, are more readily penetrated by NaOH, facilitating the generation of reactive zones. However, no consistent, obvious correlation was observed between the initial amorphous content (100%‐*X* *_C_*) and the reaction rate constant (Figure 9). Instead, the data reveal two groups with distinct surface‐amorphization behavior.

PET_25_, PET_beads_, and PET_fabric_ exhibited reaction rates nearly an order of magnitude higher than those of the remaining samples, despite their relatively low initial surface amorphous content (*X* *_C_* ^Raman^ ≈ 40%; Figure 9a). For these three samples, the rate constants correlated more strongly with the initial bulk amorphous content than the initial surface amorphous fraction (Figure 9b). Although low crystallinity is expected to promote early interface formation and accelerate depolymerization, this trend does not strictly hold for the other samples.

For PET_400_, PET_100_, PET_powder_, and PET_bottle_, the reaction rate constant showed a clearer correlation with the initial surface amorphous content than the bulk amorphous fraction. However, PET_container_ did not follow either trend, exhibiting a substantially lower rate constant despite its high initial surface amorphous content.

Given that the flake‐type samples were not fully pulverized during the first 2.5 min of milling, depolymerization during this early reaction period is expected to occur predominantly at their external surfaces rather than throughout the bulk. As a result, surface amorphization likely exerts a stronger influence on early‐stage reactivity than bulk. For this reason, surface amorphization (100%‐*X* *_C_* ^Raman^) serves as a more appropriate descriptor when evaluating early depolymerization kinetics in these systems.

**SCHEME 1:**
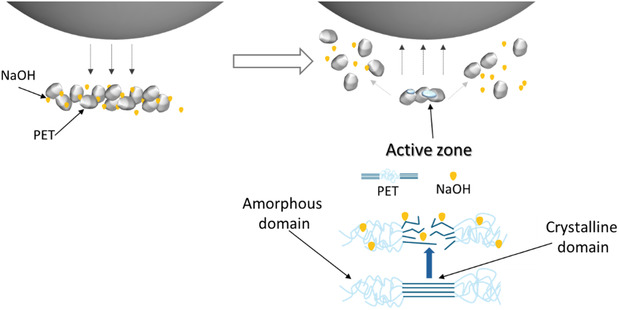
Formation of active interfaces during mechanical impact.

To investigate the reduction in crystallinity during milling, the reactivity and surface amorphization after 2.5 min of milling were analyzed (Figure 9c). While the depolymerization rate cannot be described as a simple mathematical function of the surface amorphization (100%‐*X* *_C_* ^Raman^), it is apparent that very high rates can be observed for samples with levels of surface amorphization between 65%–70%, which some samples reach after merely 2.5 min of milling (PET_25_, PET_beads_, PET_bottle_, and PET_powder_). Above this value, further reductions do not increase the reaction rate and may even hinder depolymerization due to altered mechanical behavior (see the paragraph below on the PET_container_). PET_400_ and PET_100_ did not reach this threshold within the first 2.5 min of milling, indicating that additional amorphization is required to sustain efficient interface formation. As illustrated in Figure 9d, the surface percent crystallinity of all samples decreases rapidly during the early stages of milling and subsequently approaches a plateau near 30%. A similar leveling‐off in crystallinity has been reported in prior mechanochemical studies. Bai et al. [44] showed that ball milling drives PET toward an oriented amorphous state with X_*C*_ stabilizing at ∼26%–32% independent of the initial crystallinity, and Zaker et al. [45] later demonstrated a comparable convergence to 26%–36% during milling at 30 Hz. The reduction in crystallinity appears to directly promote monomer formation, as newly amorphized domains are rapidly converted (Scheme 1).

To further support the relationship between amorphization and reactivity, PET_powder_ was pre‐milled to reduce its bulk crystallinity from 39% to approximately 30%, matching the plateau region observed across all feedstocks. Upon milling with NaOH, the pre‐milled sample exhibited a more than fivefold increase in the initial monomer formation rate (Figure 6b). Specifically, the rate constant increased from 1.25  ×  10^−4^ to 6.71  ×  10^−4^ mol/dm^3^•s (Table SI3). This behavior is consistent with the notion that enhanced amorphization facilitates more rapid monomer formation. Notably, despite its higher initial rate, the pre‐milled powder did not achieve full depolymerization within the experimental time frame. Early wax‐phase formation (at 5 min for pre‐milled powder) likely encapsulates residual PET particles in a manner that prevents their complete conversion.

In addition to crystallinity, the particle size and thickness also influenced the rate of depolymerization of the commercial samples. In the flake‐type samples, the thicker samples generally experienced slower depolymerization due to the limited exposed PET surfaces, at which reactive interfaces can be formed immediately. The conversion of thick samples can be conceptually compared to the shrinking core model, where the NaOH reacts with the outermost layer of PET on the external surface of the flakes first before converting the PET deeper inside the sample [16]. This trend was most evident when comparing the PET_400_, PET_100_, and PET_25_, with high monomer yield occurring after only 2.5 min for the PET_25_ compared to 7.5 min for both the PET_100_ and PET_400_ (Figures 7 and 8).

Comparative analysis revealed that most of the time, crystallinity had a stronger impact than thickness: PET_400_ and PET_100_ had similar surface percent crystallinity, but the quadrupled thickness of PET_400_ resulted in a reduction in the monomer formation by a factor of 1.67. PET_400_ and PET_bottle_ had similar thickness; however, the roughly doubled initial surface percent crystallinity of PET_400_ led to a reduction in the monomer formation rate by nearly 70%.

PET_fabric_ behaved similarly to the PET_25_ since the fabric fibers were roughly the same thickness as the PET_25_ (Figure 2). Commercial fabric is manufactured to have a loose structure so that the amorphous domains can be manipulated without plastic deformation [46]. Given this loose structure, there is a high initial surface area for maximum interaction between NaOH and PET, which allowed for 73% monomer yield in the first 2.5 min (Figure 8).

In the case of the particle‐like samples, there was no significant limitation due to the shape and thickness. The PET_beads_ were nearly 100 times larger than the PET_powder_ particles but had a significantly higher reaction rate (Figure 6). Compared to the particles in the powder sample, the larger and bulkier bead unexpectedly led to faster depolymerization. The PET_beads_, being more cohesive, most likely retain their structural integrity during early milling, allowing more effective collisions with generating a greater initial number of reactive interfaces and leading to more efficient amorphization. However, after the first 2.5 min of milling, the PET_beads_ experienced a slow linear increase in monomer yield for the duration of milling (Figure 6). This linear portion had a similar slope to the initial monomer yield curve of the PET_powder_ (Figure 6), suggesting that the remaining PET in the beads behaves like a powder, and the beads lose their structural integrity and cohesiveness after 2.5 min.

When considering the impact of surface percent crystallinity and thickness on the monomer formation rate of the PET_container_, there were two plausible explanations as to why the initial rate was lower than anticipated. The first is the initially surface percent crystallinity, which was initially hypothesized to promote efficient depolymerization but fell below the optimal value of 30% as discussed above. However, the very high amorphous content of this sample also results in higher ductility compared to the semi‐crystalline PET feedstocks. This likely resulted in greater flexibility and deformation of the flakes under stress prior to fracture. Thus, very amorphous PET experiences more elastic collisions, which are less effective in promoting depolymerization as evidenced by a lower rate of monomer formation from PET_container_. The second explanation for the reduction in the initial rate of monomer formation is that the thickness of the sample could reduce the accessibility of the ester bonds in PET. Since this sample was six times thicker than any other sample, the impact of the shrinking core model and surface reaction on container depolymerization was significant. Thus, thicker samples are depolymerized more slowly since the NaOH must penetrate a thicker PET core before completely converting the flake.

### 3.2 Influence of Reaction Environment on Depolymerization Efficiency

Another important factor influencing depolymerization efficiency is the reaction environment, specifically, the distribution of volume within the milling vessel. Both the fraction of empty space and the volume occupied by milling balls critically affect energy transfer during mechanochemical processing. Low void volumes restrict ball mobility, reducing collision intensity and limiting effective energy delivery to the reactants [47]. Prior studies found that an optimum balance of throughput with reduced energy consumption is obtained when a coarse feedstock is milled in a vessel filled with 25 vol% balls, 30 vol% feedstock, and a balance of void space [48, 49].

**FIGURE 10:**
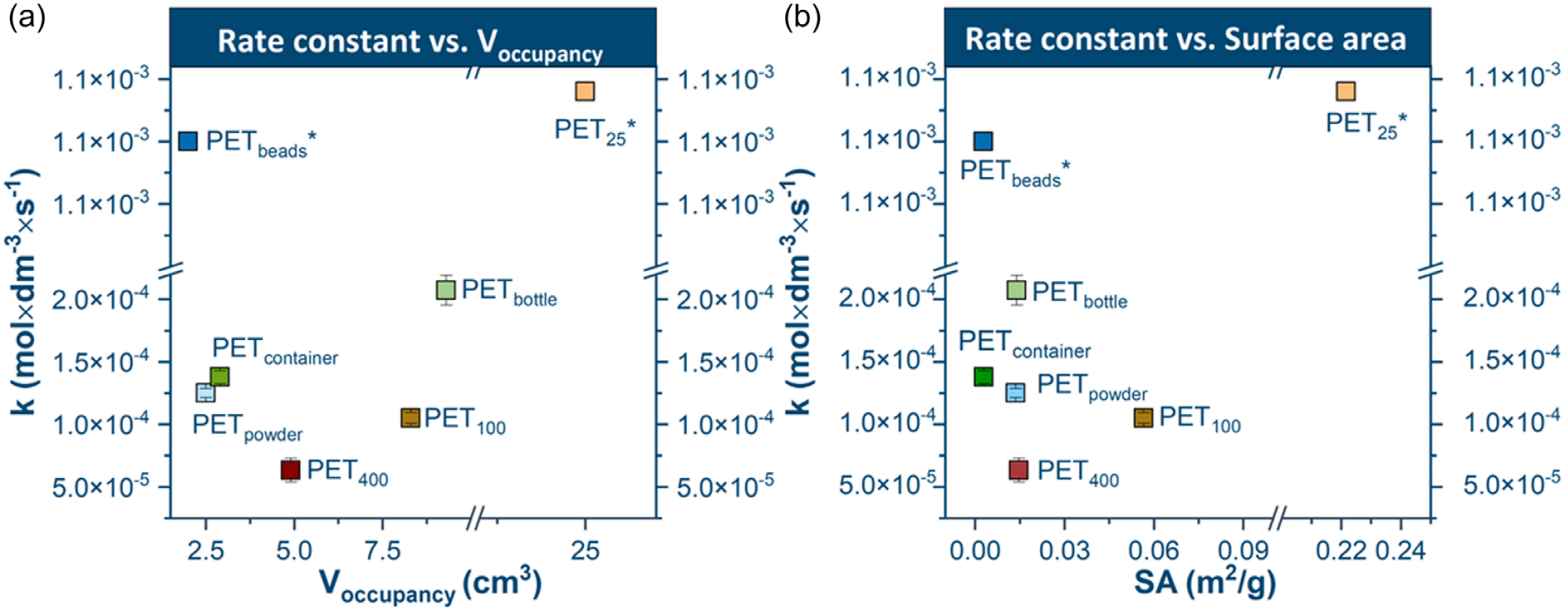
Correlation between reaction rate constant (*k*) and initial occupancy vessel volume (a) and surface area (b).

Contrary to expectations, high initial vessel feedstock occupancy did not hinder depolymerization in the study systems (Figure 10a). Samples with high initial occupancy, such as PET_25_ (25 cm^3^) and PET_fabric_ (16.2 cm^3^), exhibited rapid high monomer formation at the initial stage of the reaction (within 2.5 min). This finding indicates that ball motion was not significantly restricted. The PET_400_, PET_100_, PET_25_ samples, which shared similar shape, size, and surface percent crystallinity, demonstrated that the rate constant for the depolymerization reaction even increased (*k* = 6.3 × 10^−5^, 1.05 × 10^−4^, and 1.12 × 10^−3^ mol/dm^3^·s respectively) with increasing initial occupancy (4.9 for PET_400_, 8.3 for PET_100_, and 25 cm^3^ for PET_25_). Additionally, the PET_25_ was the only one of these three samples to reach the optimal crystallinity value after 2.5 min of milling, indicating that high occupancy did not hinder the effectiveness of collisions. This trend extended to PET_bottle_ and PET_container_. Thus, it is concluded that the film samples are rapidly pulverized and that the thickness of the film has a much larger effect on depolymerization kinetics than any restriction of the ball motion in the early moments of the milling process.

The surface accessibility is often assumed to enhance reaction kinetics, but the data (Figure 10b) reveal substantial deviations from this expectation. Notably, PET_beads_ characterized with the smallest (0.003 m^2^/g) and PET_25_ with the largest (0.23 m^2^/g) initial surface area exhibited similar reaction rate constants, despite their morphological differences. Additionally, PET_400_, which had one of the lowest surface areas, showed the lowest reaction rate, while PET _powder_ with an almost identical surface area to PET_400_ achieved significantly faster depolymerization. These inconsistencies suggest that the initial surface area alone cannot explain the observed kinetic behavior either. It is important to note that surface area is a dynamic property during milling; while particle size reduction typically increases surface area, subsequent sintering or agglomeration of fine particles can lead to its decrease, complicating its use as a reliable descriptor of reactivity.

While the high vessel occupancy of thin PET_25_ and PET_100_ films did not affect the initial depolymerization rate, achieving quantitative monomer yield was harder from these samples. These feedstocks are converted into the wax phase sooner in the process (Table SI4), and it appears that a wax layer encapsulates some of the remaining PET domains in such a way that they cannot be converted as readily. Thus, PET_25_ and PET_100_ failed to reach 100% monomer yield within 20 min of milling, while PET_400_ did.

## 4 Conclusion

Mechanochemical depolymerization of different consumer‐grade PET feedstocks with NaOH was successful for all samples and did not require any specific sample preparation or pretreatment. High monomer yields are reached in 20 min at ambient reaction conditions, supporting the potential for this depolymerization method at large scales.

Crystallinity is considered a key determinant of depolymerization efficiency, with amorphous domains promoting faster interface formation and monomer release. Samples with low initial crystallinity (PET_container_ and PET_bottle_) are converted into monomers faster than those that require amorphization before depolymerization (PET_400_ and PET_100_). Specifically, a surface percent crystallinity of approximately 30% appears to be optimal for this reaction because further reduction in crystallinity may hinder depolymerization due to increased ductility and reduced fracture efficiency during milling. This phenomenon was seen clearly with the PET_container_, which had the lowest initial surface percent crystallinity but a slower depolymerization rate than the PET_bottle._

In addition to crystallinity, flake thickness and morphology significantly affect reaction rates, especially for the flake‐type samples, where accessibility is limited. Thinner samples experience faster depolymerization since PET is more easily accessible as the reaction proceeds according to the shrinking core model. Despite the influence of thickness, the particle‐like samples, specifically the PET_beads_, show unexpected behavior, with larger particles sometimes depolymerizing at faster rates due to cohesive structure and effective collisions. The PET_beads_, which had a more cohesive particle shape than the PET_powder_, exhibited faster depolymerization since collisions with this cohesive structure were more efficient in depolymerizing the PET.

While thickness and morphology have a great impact on reaction rates, surface area and vessel occupancy were both insufficient in predicting kinetic behavior. The surface area is dynamically altered during milling through particle size reduction and sintering, making it a difficult factor to track during the reaction duration. Although vessel occupancy was hypothesized to hinder depolymerization due to ball motion restrictions, no correlation between initial occupancy and depolymerization rate is seen for the PET samples studied here because the most voluminous samples were pulverized sufficiently quickly, and the benefits of their lower thickness overcompensated any potential effects of occupancy.

Overall, depolymerization efficiency is multifactorial, requiring consideration of crystallinity, morphology, milling dynamics, and reaction environment for accurate prediction and optimization of the milling process. Given the successes of the mechanochemical depolymerization of PET using a NaOH catalyst in a ball mill, potential implementation of this method at an industrial scale could contribute to increasing the sustainability of polymer upcycling because of its mild reaction conditions and minimal solvent use.

## 5 Experimental Methods

### 5.1 Materials

Eight different feedstocks were tested. The PET pellets with an average diameter of 300 μm were obtained from PolyQuest Inc. The three PET films of thicknesses 0.004 in [101.6 μm] (PET_400_), 0.001 in [25.4 μm] (PET_100_), and 0.0005 in [6.35 μm] (PET_25_) were purchased from McMaster‐Carr. The fabric, food container, and bottle are all post‐consumer plastics and were obtained from retail grocery stores. Sodium hydroxide (>97%) crystals were purchased from Sigma–Aldrich.

### 5.2 Milling Procedure

A similar milling procedure as described by Tricker et al. was followed [16]. 1.0 g of the chosen PET feedstock was added to the 25 mL stainless steel vessel along with 0.42 g of the NaOH crystals. A 20 mm diameter stainless steel ball was added to the vessel before closing it with an O‐ring and placing it in a Retsch MM400 vibratory ball mill. The samples were milled for 20 min at a 30 Hz milling frequency, and every 2.5 min, a small sample was taken from the milling vessel to track yield to monomer production over time.

The PET beads and pellets were taken as purchased and added directly to the milling vessel. The PET films, fabric, bottle, and food container were cut into roughly 0.5 by 0.5 cm flakes before being milled. Using these approximate flake dimensions along with the density of each sample, the external surface area, *SA*_*E*_, was calculated (Equation (3)). External surface area was defined as the surface area of each flake (Equation (1)) multiplied by the number of particles in each flake and normalized by the 1.0 g sample mass. Total number of particles in each flake, *n*_particles_, was determined by multiplying the flake volume by the density of the feedstock and dividing this by the total 1.0 g sample mass (Equation (2)). The densities (*ρ*) of the feedstock samples are summarized in Table SI5. The equations below demonstrate how the flake external surface area was calculated, where *t* is the thickness of each sample, and L is the length of the flake.

(1) $${\mathrm{SA}}_{\text{flake}}=\left(L\times L\times 2\right)+(L\times t\times 4)$$

(2) $$n_{\text{particles}}=\frac{1.0\;g\;\text{sample}}{V_{\text{flake}}\times \text{ρ}}$$

(3) $${\mathrm{SA}}_{E,\text{flake}}={\mathrm{SA}}_{\text{flake}}\times n_{\text{particles}}$$

The external surface area of the powder and beads was also computed by normalizing the surface area of all the particles in one particle by the total mass of 1.0 g (Equation (7)). The surface area was calculated using the given diameter of the powder particles and measured long and short diameters of the beads, which were *D*_*L*_ = 3.25 mm and *D*_*S*_ = 2.26 mm respectively (Equations (4) and (5)). The total number of particles was determined using the same procedure highlighted above (Equations (6)). The thickness of the beads was also computed, which was 2.24 mm.

(4) $${\mathrm{SA}}_{\text{powder}}=4\times \text{π}\times {\left(\frac{D}{2}\right)}^{2}$$

(5) $${\mathrm{SA}}_{\text{beads}}=\text{π}\times \left(\frac{D_{L}}{2}\right)\times \left(\frac{D_{S}}{2}\right)\times t$$

(6) $$n_{\text{particles}}=\frac{1.0\;g\;\text{sample}}{V_{\text{flake}}\times \text{ρ}}$$

(7) $${\mathrm{SA}}_{E,\text{powder or beads}}={\mathrm{SA}}_{\text{powder or beads}}\times n_{\text{particles}}$$

PET powder pre‐milled: To achieve a certain level of amorphousness in the starting material, 2.0 g of the PET powder was pre‐milled in a SS316 stainless steel vessel using one 20 mm stainless steel ball, under the frequency of 30 Hz and a duration of 60 min, also using the MM400. The processing of mechanochemical alkali‐hydrolysis of the pre‐milled samples follows the aforementioned procedure as for all other samples.

### 5.3 Raman Spectroscopy

A Renishaw inVia confocal Raman spectro‐microscope was used, which included a temperature‐controlled CCD camera. The spectra were acquired using a 785 nm high‐power near‐infrared diode laser with an objective of 20x magnification. The spectra were measured at five discrete locations on the powdered surface with 15 accumulations at each site. The spectra were measured in static mode with a 1.5 s exposure time and laser power of 50% of the total laser power. Baseline correction and normalization relative to the band at 1615 cm^−1^ were conducted using the Windows‐based Raman Environment (WiRE) software.

The surface percent crystallinity was established based on the method proposed by Bouita et al. [31]. The normalized spectra were deconvoluted using the Python script. The peak integral intensities of the bands at 1721, 1727 , and 1733 cm^−1^ were collected for each of the five sampling locations at each time. Then, using the formula below, the surface percent crystallinity (*X* *_C_* ^Raman^) was calculated by dividing the area under the representative peak of crystalline PET at 1727 cm^−1^ by the sum of the areas under the peaks representing amorphous and crystalline phases at 1721, 1733, and 1726 cm^−1^. This crystalline to amorphous ratio was calculated at each of the five discrete sampling locations and averaged.

(8) $$X_{C}^{\text{Raman}}=\frac{1727\;{\mathrm{cm}}^{-1}\;\text{integral peak intensity}}{1721\;{\mathrm{cm}}^{-1}\;\text{integral peak intensity}+1733\;{\mathrm{cm}}^{-1}\;\text{integral peak intensity}+1727\;{\mathrm{cm}}^{-1}\;\text{integral peak intensity}}\cdot 100\%$$

### 5.4 HPLC

HPLC analysis was conducted using the same method as Tricker et al. to analyze the yield of monomer throughout the depolymerization process [16]. For each sample, between 1 and 10 mg were diluted with 1 mL of deionized (DI) water in a centrifuge tube (solution A). This sample was then vortexed and sonicated for 30–45 min to maximize dissolution. The sample was then placed to rest overnight to allow any insoluble residues to settle. Next, 10 μL of solution A was pipetted into another centrifuge tube along with 1 mL of DI water (solution B). Then, 100 μL of solution B was pipetted into a third centrifuge tube along with 600 μL of 0.1 wt% H_3_PO_4_ in water and 300 μL of MeOH. This final solution was filtered through a 0.2 μm poly(tetrafluoroethylene) (PTFE) syringe filter into an HPLC sample vial for analysis. The yield to monomer was calculated as the ratio of moles of the product to moles of initial PET in the reaction (Equation (9)).

(9) $$Y_{\text{monomer}}=\frac{n_{\text{monomer},f}}{n_{\mathrm{PET},0}}$$

### 5.5 SEM

A Thermo Axia Variable Pressure Model with a thermionic tungsten filament source and Everhart–Thornley detector was used to conduct SEM imaging. Carbon tape was used to mount the samples, which were analyzed under high vacuum. Operating voltage ranged from 3.00 to 10.00 kV while the working distance ranged between 7.5–10.7 mm. Scales for the images range from 100 to 2 mm.

### 5.6 XRD

The analysis of crystallinity of Na_2_TPA was performed on a Rigaku Miniflex Powder diffractometer at room temperature utilizing Cu K_*α*_ radiation (40 kV; *λ * = 1.54 Å). Diffractograms were collected in a 2*θ* range of 5°–70° with a scanning speed of 5° min^−1^ without rotation. The degree of crystallinity was calculated using the ratio of the integrated area under the crystalline peaks to the total integrated area of the diffraction pattern.

### 5.7 N_2_ Pycnometry

The material density was measured by gas displacement pycnometry using a Micromeritics AccuPyc II 1345 instrument with ultra‐high‐purity nitrogen as the displacement medium. Approximately 1 g of the original sample was cut, weighed, and placed into a 10 cm^3^ sample cell. Each measurement consisted of ten initial purge cycles followed by five analysis cycles at a fill pressure of 19.5 psi to ensure gas purity and stable measurement conditions.

### 5.8 DSC

The degree of crystallinity of the materials was quantified using a TA DSC 250. Measurements were performed using standard aluminum pans and an empty pan as a reference. Samples were heated from room temperature to 300°C at 10°C min^−1^ under a nitrogen purge of 50 mL min^−1^. The specific heat capacity as a function of temperature was obtained from the normalized heat flow signal. For each sample, the crystallization exotherm and the melting endotherm were integrated to obtain the enthalpy of cold crystallization (*Δ* *H*_cc_) and the enthalpy of melting (Δ*H*_m_), respectively. The degree of crystallinity at room temperature was calculated from the following equation:

(10) $$X_{c}^{\mathrm{DSC}}=\frac{\left|\text{Δ}H_{m}\right|-\left|\text{Δ}H_{\mathrm{cc}}\right|}{\text{Δ}H_{f}^{100}}$$

## Supporting Information

Additional supporting information can be found online in the Supporting Information section. **Supporting** **Fig.** **S1:** Images of feedstocks before milling, with PET_fabric_, PET_100_, PET_bottle_, PET_400_, PET_container_, PET_powder_, and PET_beads_ in 25 mL glass containers (a) and PET_25_ in the 25 mL milling vessel (b). **Supporting Fig.** **S2:** Picture of PET_beads_ during mechanochemical alkali‐depolymerization collected every 2.5 min. **Supporting Fig.** **S3:** Concentration of Na_2_TP obtained from depolymerization of PET_powder_ (a), PET_400_ (b) PET_100_ (c), PET_bottle_ (d), PET_container_ (e), PET_25_ (f) PET_beads_ (g) and PET_fabric_ (h) and PET_pre‐milled_ powder with intiall *X*_*C*_^DSC^ = 30% (i) over time. **Supporting Fig.** **S4:** DSC thermogram of PET_powder_. **Supporting Fig.** **5:** DSC thermogram of PET_beads_. **Supporting Fig.** **S6:** DSC thermogram of PET_400_. **Supporting Fig.** **S7:** DSC thermogram of PET_100_. **Supporting Fig.** **8:** DSC thermogram of PET_25_. **Supporting Fig.** **S9:** DSC thermogram of PET_bottle_. **Supporting Fig.** **S1** **0:** DSC thermogram of PET_container_. **Supporting Fig.** **S11:** DSC thermogram of PET_fabric_. **Supporting Fig.** **S1** **2:** DSC thermogram of PET_pre‐milled_. **Supporting** **Table S1:** Occupancy volumes computed for all samples milled up to 15 min. **Supporting Table** **S2:** The percent crystallinity obtained from DSC and Raman analyses for all samples. **Supporting Table** **S3:** Initial rates of reaction and corresponding *R* ^2^ values. **Supporting Table** **S4:** Time of sample transition into homogeneous wax phase. **Supporting Table** **S5:** The densities (*ρ*) and standard deviations (*σ*) of the feedstock samples. **Supporting Table** **S6:** The percent crystallinity obtained from Raman analyses computed for all samples milled up to 12.5 min.

## Funding

This study was supported by U.S. National Science Foundation (Grant 2028998).

## Conflicts of Interest

The authors declare no conflicts of interest.

## Supporting information

Supplementary Material

## Data Availability

The data that support the findings of this study are available from the corresponding author upon reasonable request.
