# Supplementary material for: Mechanochemical Depolymerization of PET: Kinetic Studies on Alkaline Hydrolysis of Commercial Feedstocks
Source: ChemSusChem. 2026 Feb 12;19(3):e202502416. doi: 10.1002/cssc.202502416 (PMC12900266; doi:10.1002/cssc.202502416)
Supplement: Supplementary file 1 — Supplementary Material [file CSSC-19-e202502416-s001.pdf]

Supplementary Information

Mechanochemical Depolymerization of PET: Kinetic Studies on Alkaline Hydrolysis of Commercial Feedstocks

Kinga Gołąbek§ , Lauren R. Mellinger§ , Shanell T. Bush, Erin V. Phillips, Georgios A. Marinis, Van Son Nguyen, Jouke van Westrenen and Carsten Sievers*

§ Equal contribution

School of Chemical & Biomolecular Engineering
Georgia Institute of Technology
Atlanta, Georgia, 30332, United States
E-mail: carsten.sievers@chbe.gatech.edu

A known mass of powder was transferred into a cylindrical container of known diameter (D). After gently tapping the container to allow the sample to settle without over-packing, the height of the powder bed ($h_{measured}$) was measured with a ruler. The sample volume was then calculated using equation below.

$$V_{occupancy}=\pi\left(\frac{D}{2}\right)^2 \times h_{measured}$$

Table SI1. Occupancy volumes computed for all samples milled up to 15 minutes.

Time (min)	PET_{powder} (cm^3)	PET_{beads} (cm^3)	PET_{400} (cm^3)	PET_{100} (cm^3)	PET_{25} (cm^3)	PET_{bottle} (cm^3)	$PET_{container}$ (cm^3)	PET_{fabric} (cm^3)
0	2.5	2.0	4.9	8.3	25	9.3	2.9	16.2
2.5	2.9	2.9	6.9	7.9	3.9	2.9	2.7	2.0
5	2.5	2.0	3.9	7.4	3.4	2.7	2.5	1.5
7.5	2.5	1.7	3.9	2.9	2.7	2.5	2.7	ND
10	3.2	1.7	2.9	2.5	2.9	2.2	2.0	ND
12.5	3.4	1.7	2.9	3.4	2.7	2.0	2.0	ND
15	ND	2.5	2.9	3.4	2.2	2.5	2.5	ND

*ND means that the sample already began to transition into the wax phase and a correct occupancy was unable to be computed.

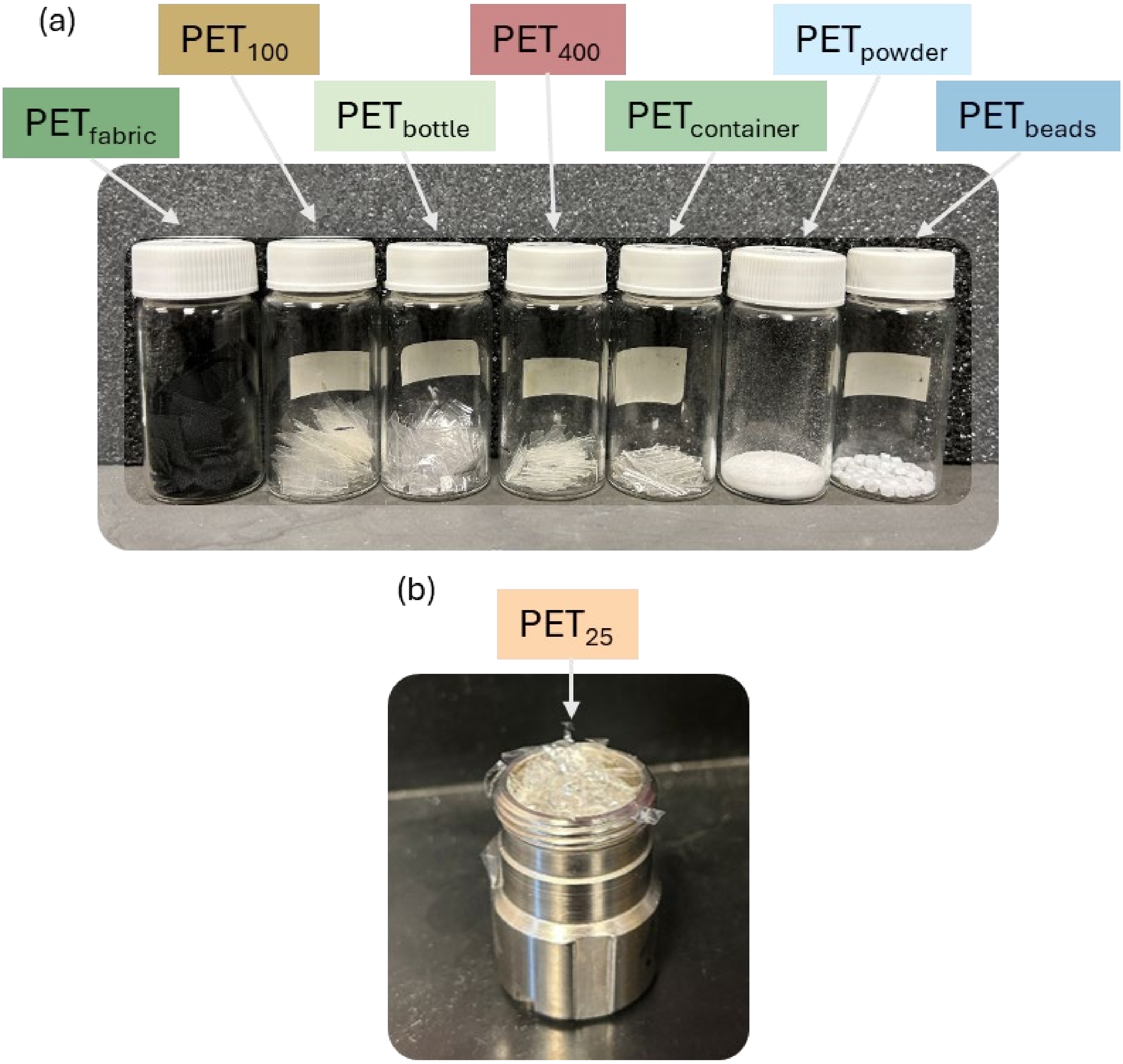

Figure SI1. Images of feedstocks before milling, with PET_{fabric}, PET_{100}, PET_{bottle}, PET_{400}, $PET_{container}$, PET_{powder}, and PET_{beads} in 25 mL glass containers (a) and PET_{25} in the 25 mL milling vessel (b).

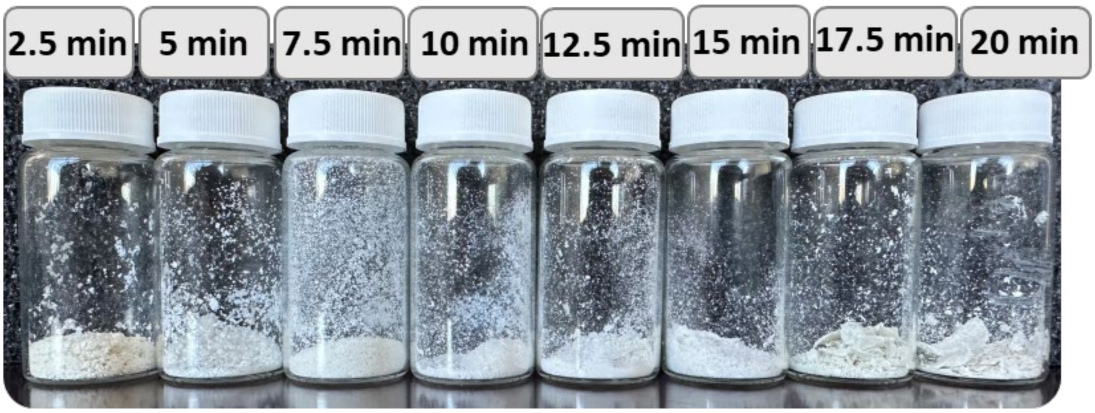

Figure SI2. Picture of PET_{beads} during mechanochemical alkali-depolymerization collected every 2.5 minutes.

Table SI2. The percent crystallinity obtained from DSC and Raman analyses for all samples.

Sample	X_c^{DSC}	X_c^{Raman}
PET_{powder}	39%	40%
$PET_{pre\text{-}milled}$	30%	29%
PET_{beads}	41%	40%
PET_{400}	28%	45%
PET_{100}	29%	43%
PET_{25}	32%	41%
PET_{bottle}	22%	31%
$PET_{container}$	6%	19%
PET_{fabric}	39%	ND

Kinetic Model Calculations.

Kinetic analyses of solid–solid or solid–liquid transformations are typically based on the initial linear region of product formation, where diffusion limitations and phase-transition effects are minimal. Following this convention, the kinetic fitting in this work was performed using only the early-time data points prior to the wax-phase transition of each PET feedstock.

For PET_{powder}, the first six data points of the Na_2-TPA concentration vs. time curve were used for fitting because the transition from powder to wax occurred at 12.5 minutes, after which the monomer concentration increased sharply (Figure 5). The same six initial points were used for PET_{bottle} and $PET_{container}$, as these samples exhibited comparable phase-transition times.

In contrast, PET400 and PET100 showed a more gradual and heterogeneous wax-phase transition, where the inflection point corresponded to a mixture of residual powder and wax agglomerates. This heterogeneity hindered reliable model fitting across the full early-time range. Therefore, the kinetic fitting was performed using only the points clearly preceding the onset of transition—five points for PET100 and four points for PET400.

For PET25, PET_{beads}, and PET_{fabric}, the initial monomer formation rates were substantially higher (Figures 5 and 6). Because the early portion of their concentration–time curves contained only a few measurable points before rapid plateauing, the initial rates were determined by linear fitting of the first two points for PET25 and PET_{beads}, and the first three points for PET_{fabric} (Figure SI3). Although fitting a line to only two or three points may overestimate the absolute initial rate, this approach provides a consistent and sufficiently accurate relative measure for comparing monomer formation rates across all flake-type feedstocks.

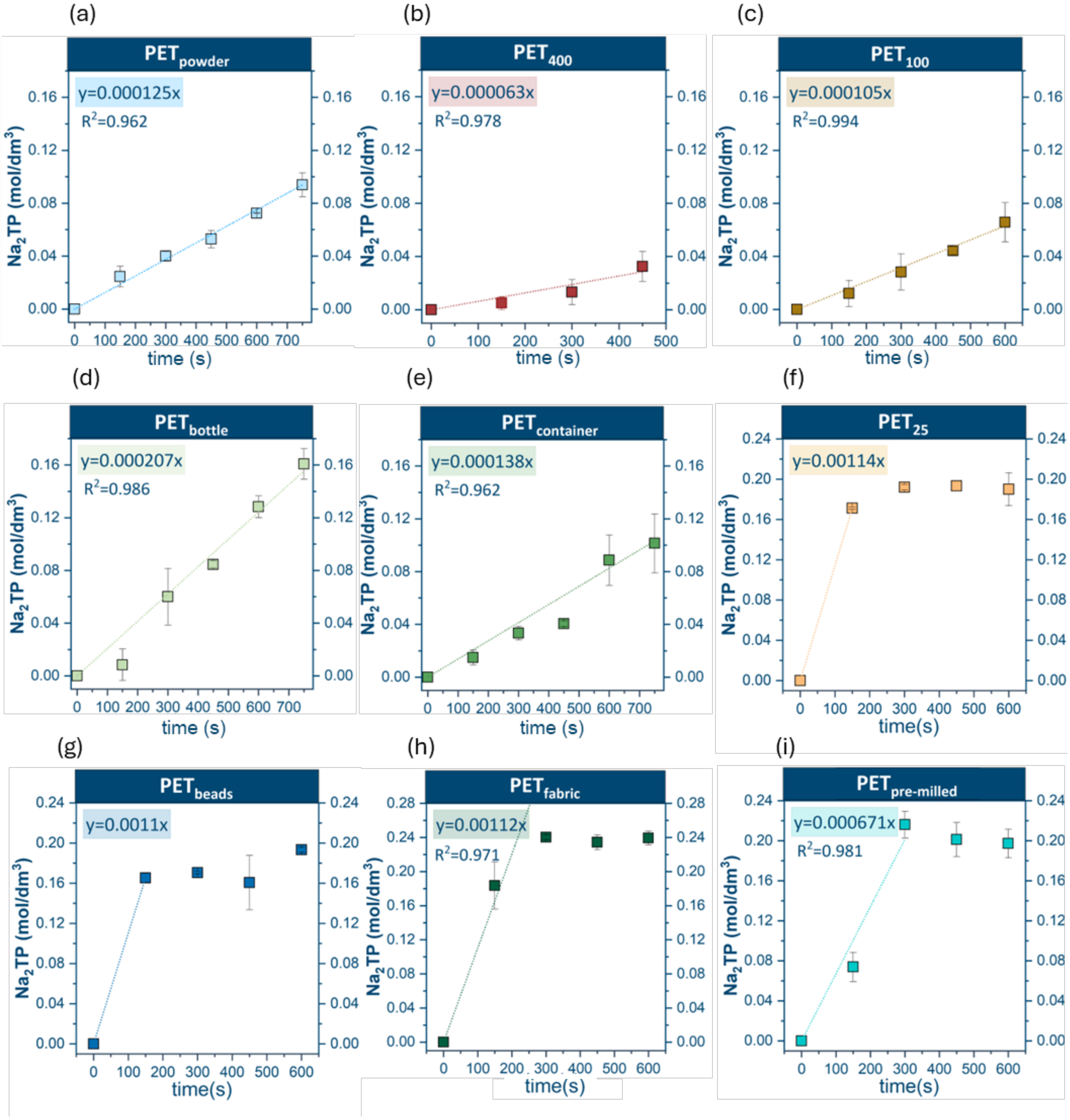

Figure SI3. Concentration of Na_2TP obtained from depolymerization of PET_{powder} (a), PET_{400} (b) PET100 (c), PET_{bottle} (d), $PET_{container}$ (e), PET_{25} (f) PET_{beads} (g) and PET_{fabric} (h) and $PET_{pre\text{-}milled}$ powder with intiall X_c^{DSC} =30% (i) over time.

Table SI3. Initial rates of reaction and corresponding R^2 values.

	PET_{powder}	$PET_{pre\text{-}milled}$	PET_{400}	PET_{100}	PET_{bottle}	$PET_{container}$	PET_{fabric}	PET_{25}	PET_{beads}
Initial Rate (mol/dm^3-•s)	$1.25x10^{-4}$	$6.71x10^{-4}$	$6.3x10^{-5}$	$1.05x10^{-4}$	$2.07x10^{-4}$	$1.38x10^{-4}$	$1.12x10^{-3}$	$1.14x10^{-3}$	$1.11x10^{-3}$
R^2 Value	0.962	0.981	0.978	0.994	0.986	0.962	0.971	-	-

Table SI4. Time of sample transition into homogeneous wax phase.

	PET_{powder}	$PET_{pre\text{-}milled}$	PET_{400}	PET_{100}	PET_{bottle}	$PET_{container}$	PET_{25}	PET_{beads}	PET_{fabric}
Wax Phase Transition [min]	12.5	5	15	17.5	17.5	17.5	17.5	17.5	5

Table SI5. The densities (ρ) and standard deviations (σ) of the feedstock samples.

Sample	**ρ[g/cm^3]**	**σ[g/cm^3]**
PET_{powder}	1.472	±0.009
PET_{beads}	1.395	±0.008
PET_{400}	1.402	±0.004
PET_{100}	1.406	±0.004
PET_{25}	1.425	±0.003
PET_{bottle}	1.369	±0.014
$PET_{container}$	1.337	±0.003
PET_{fabric}	1.509	±0.034

Table SI6. The percent crystallinity obtained from Raman analyses computed for all samples milled up to 12.5 minutes.

Time (min)	PET_{powder}	$PET_{pre\text{-}milled}$	PET_{beads}	PET_{400}	PET_{100}	PET_{25}	PET_{bottle}	$PET_{container}$
0	40.06%	28.12%	39.89%	45.30%	42.51%	41.20%	30.80%	18.95%
2.5	32.94%	ND	29.26%	41.78%	41.72%	29.61%	30.68%	18.60%
5	27.60%	ND	27.51%	28.29%	40.98%	28.41%	28.42%	16.72%
7.5	25.62%	ND	27.79%	30.82%	42.40%	27.76%	25.37%	16.72%
10	25.76%	ND	27.70%	30.13%	30.77%	27.10%	29.39%	16.72%
12.5	27.97%	ND	27.28%	29.61%	26.97%	28.27%	25.27%	16.66%

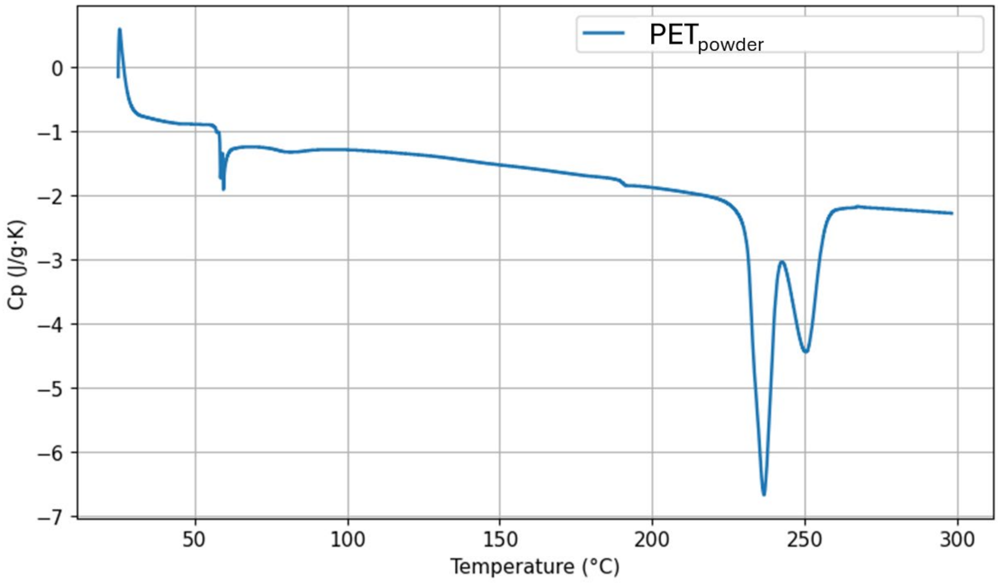

Figure SI4. DSC thermogram of PET$_{powder}$.

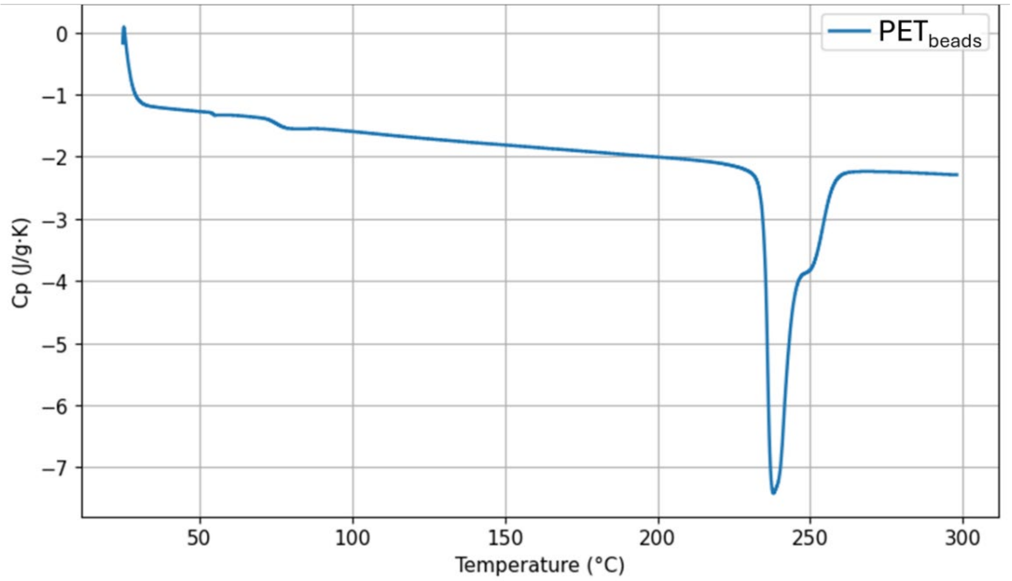

Figure SI5 DSC thermogram of PET$_{beads}$.

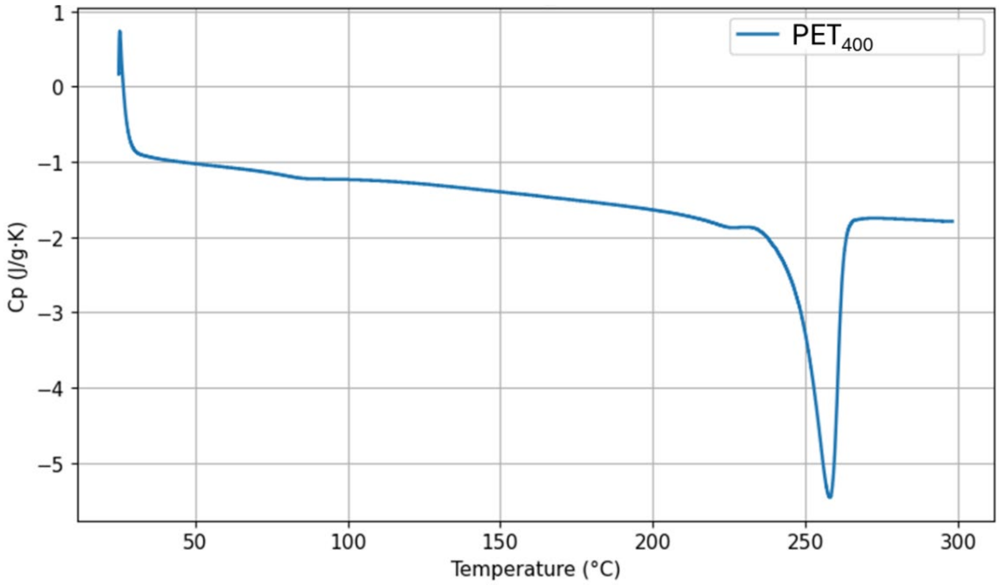

Figure SI6. DSC thermogram of PET_{400}.

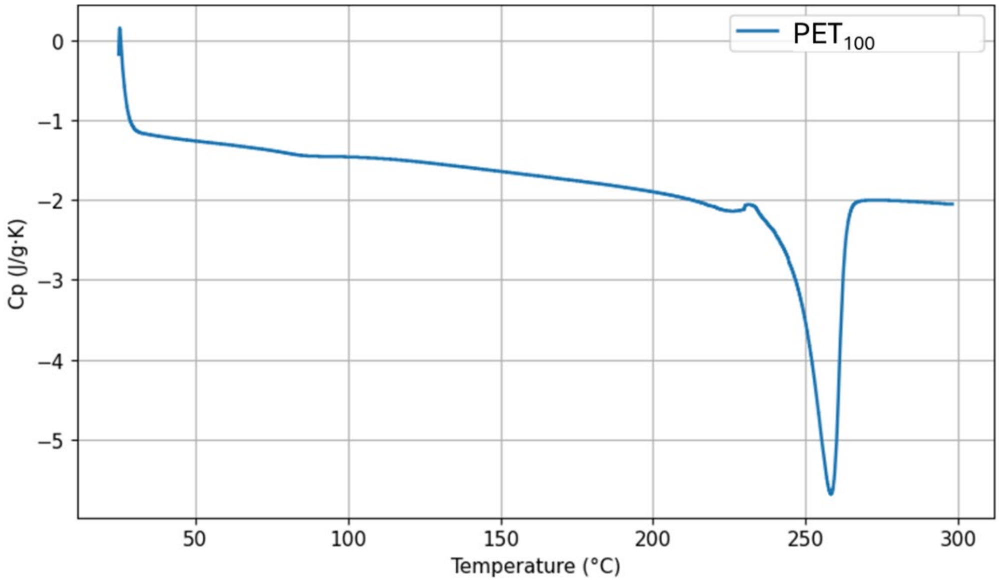

Figure SI7. DSC thermogram of PET_{100}.

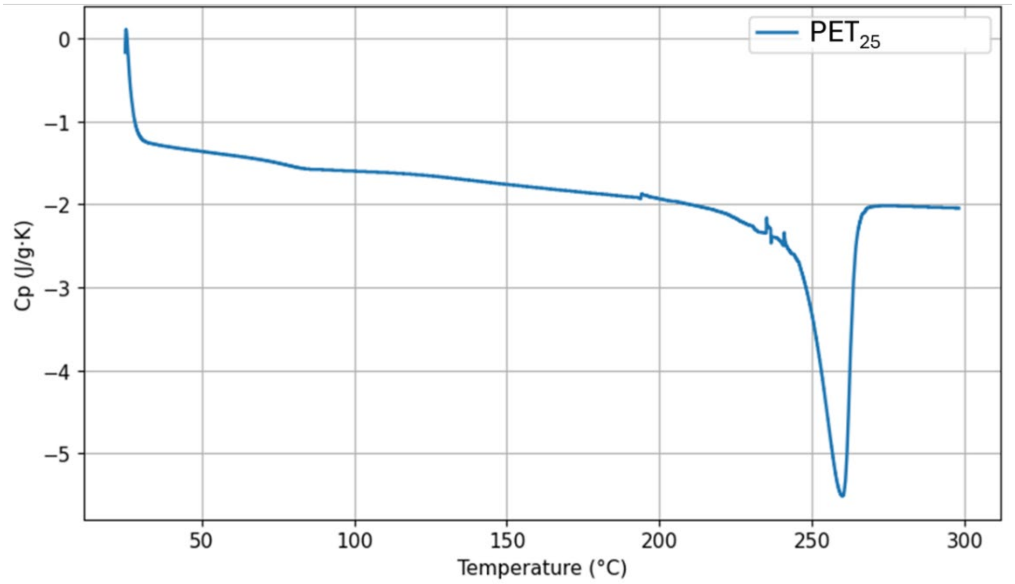

Figure SI8. DSC thermogram of PET_{25}.

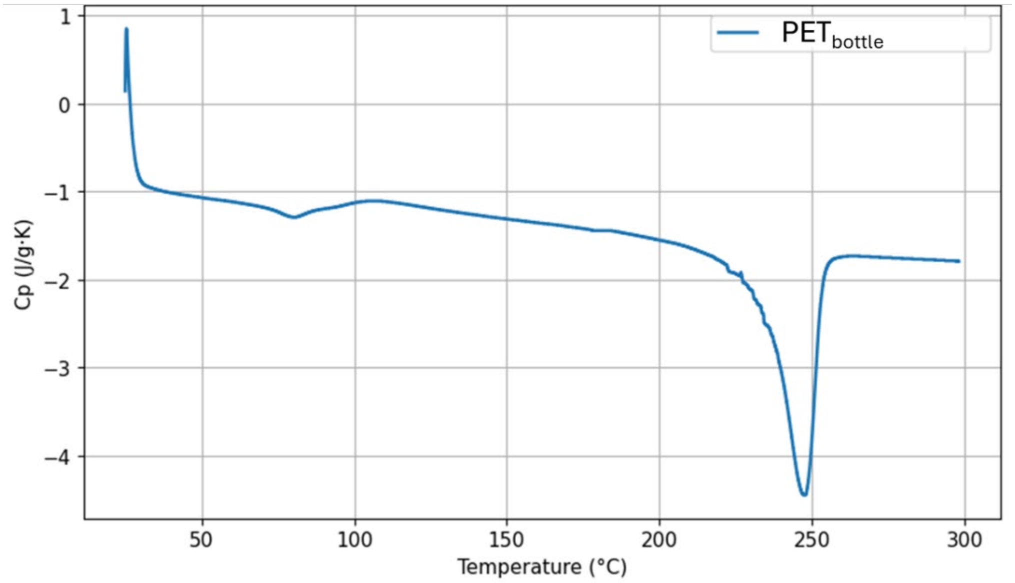

Figure SI9. DSC thermogram of PET_{bottle}.

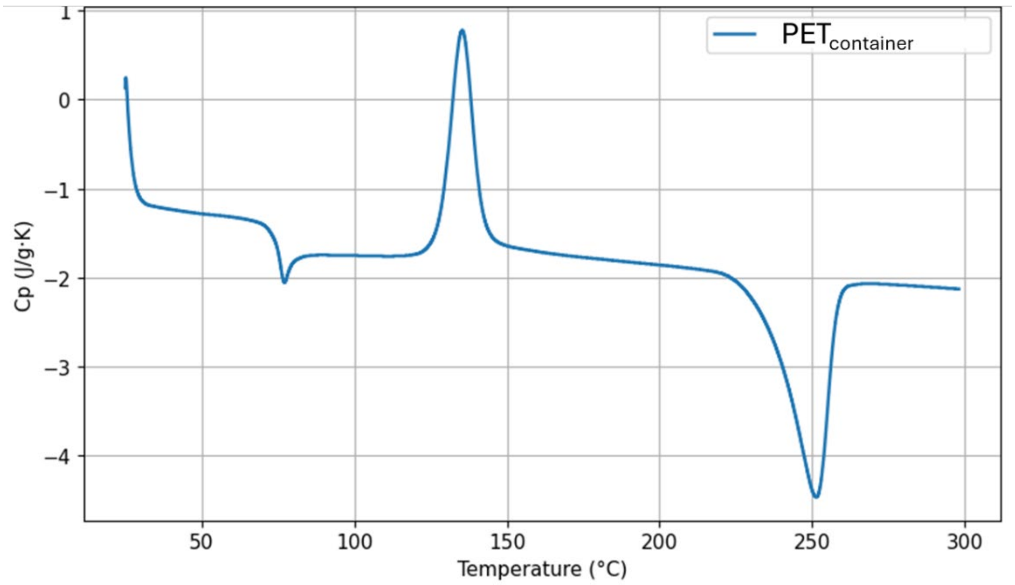

Figure SI10. DSC thermogram of PET$_{container.}$

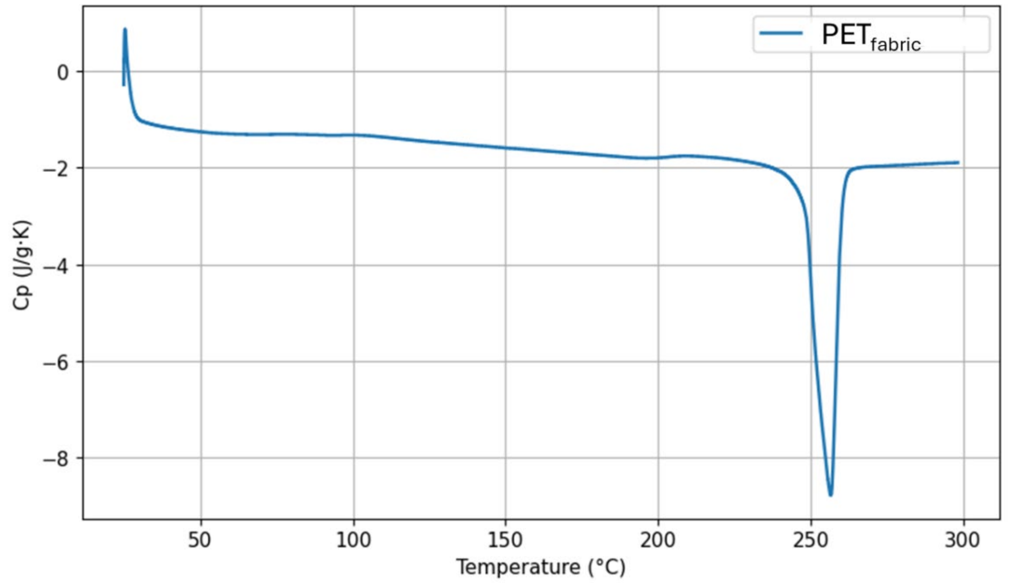

Figure SI11. DSC thermogram of PET$_{fabric.}$

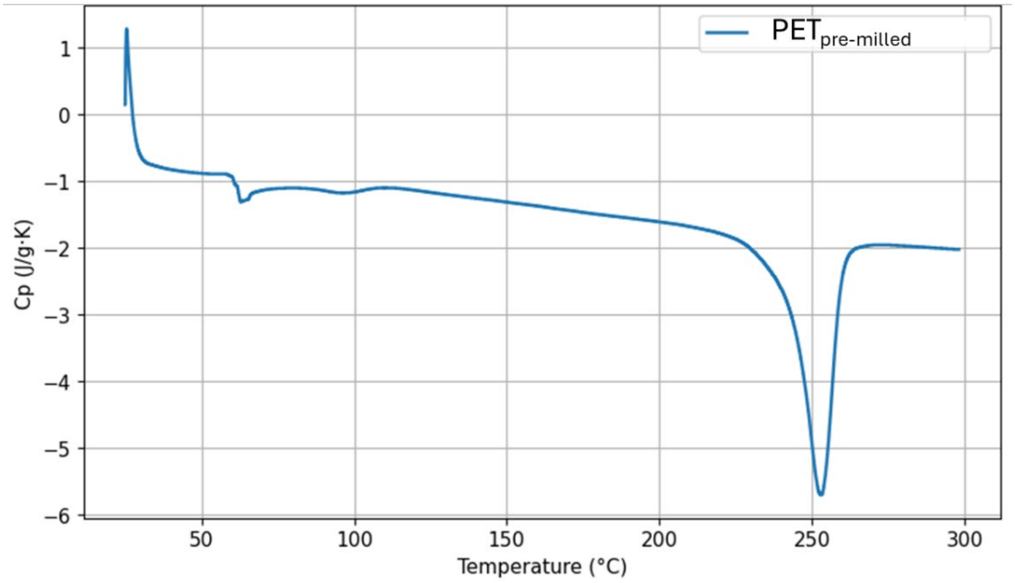

Figure S12. DSC thermogram of PET$_{pre\text{-}milled}$.
